# Toward Robust and Reliable Muscle Synergy Analysis: A Methodological Survey of Synergy Extraction

**DOI:** 10.3390/s26216700

**Published:** 2026-10-23

**Authors:** Yaru Chen, Xiaohong Wang, Yongxuan Wang, Pengju Xie, Liangchi Zhan, Haibin Liu, Hongyu Yin, Rong Liu

**Affiliations:** 1 Central Hospital of Dalian University of Technology, Dalian 116021, China; chen0123@mail.dlut.edu.cn (Y.C.); xhwang75@163.com (X.W.); yinhongyu19871987@163.com (H.Y.); 2 Liaoning Key Laboratory of IC & BME System, School of Biomedical Engineering, Dalian University of Technology, Dalian 116024, China; 13974265797@163.com (P.X.); liangchi_zhan@mail.dlut.edu.cn (L.Z.); 3 School of Information Engineering, Dalian University, Dalian 116622, China; wangyongxuan@dlu.edu.cn; 4 School of Kinesiology and Health Promotion, Dalian University of Technology, Dalian 116622, China; liuhaibin@dlut.edu.cn

**Keywords:** muscle synergies, neuromuscular controllers, extraction methodologies, robustness, reliability

## Abstract

Muscle synergy analysis has been widely applied in neuroscience, biomechanics, rehabilitation, and robotics. However, the reliability of muscle synergy extraction remains a critical issue, as results are highly sensitive to variations in experimental design, surface electromyography preprocessing, factorization algorithms, and the determination of the number of synergies. This narrative methodological review examines the key factors that influence the robustness and reliability of muscle synergies within the framework of limb neuromechanical controllers across anatomical regions and task contexts. By analyzing the current state of research in this field, we first summarize the conceptual and methodological foundations of muscle synergy analysis and then critically examine the factors that influence its robustness and reliability. This review provides practical recommendations to enhance methodological rigor, including greater methodological harmonization and transparent reporting of experimental protocols, the use of musculoskeletal simulation to evaluate muscle selection strategies, and the refinement of synergy extraction techniques. These insights aim to improve the reliability and robustness of muscle synergy analysis and promote its application in robotics, human–machine interfacing, and computational motor control.

## 1. Introduction

Surface electromyography (sEMG) is widely used in control applications because it provides a noninvasive, wearable, high-temporal-resolution means of capturing neuromuscular intent, enabling proportional and intuitive interfaces for limb assistive technologies [1]. In recent decades, sEMG has been increasingly applied in rehabilitation and clinical settings, including post-stroke and spinal cord injury therapy, orthopedic and neuromuscular disorder treatment, myoelectric prostheses, and robotic exoskeletons [2]. These human–machine interfaces (HMIs) and exoskeleton systems can support motor rehabilitation, improve functional independence, and augment human capabilities across clinical, industrial, and daily-life contexts.

Nevertheless, sEMG-based control faces several well-known challenges, including high-dimensional and redundant multimuscle signals; low signal-to-noise ratio (SNR) caused by cross-talk and motion artifacts; pronounced nonstationarity and inter-/intra-session drift driven by electrode shift, skin impedance changes, and fatigue; and strong subject- and task-dependence that complicate cross-user and cross-task generalization [3]. As the number of controlled degrees of freedom (DOFs) increases and tasks become more diverse, control pipelines must process larger numbers of channels while adapting to time-varying signal characteristics, thereby increasing computational burden and reducing generalizability. Among these challenges, achieving robust and reliable musculoskeletal control under complex, disturbance-rich conditions with pronounced interindividual variability remains a primary bottleneck.

Given that simultaneous multi-DOF control using sEMG depends on the identification of complex intermuscular interactions, a principled way to mitigate these difficulties is to leverage muscle synergies. Muscle synergies are commonly described as low-dimensional modules that capture coordinated muscle-activation patterns and provide an interpretable representation of neuromuscular control [4]. The synergy framework addresses a fundamental problem in motor control: how the central nervous system (CNS) manages the massive redundancy of the musculoskeletal system. Rather than commanding individual muscles independently, the CNS has been hypothesized to activate groups of muscles as functional and coordinated modules [5,6,7,8,9,10]. In the commonly used matrix formulation, the preprocessed sEMG data ***X*** ∈ ℝ^(*m*×*N*)^, where *m* denotes the number of recorded muscles and *N* denotes the number of sampled time points, is approximated as

***X*** ≈ ***WC*** + ***e*** (1)

Here, ***W*** ∈ ℝ^(*m*×*k*)^ contains time-invariant muscle-weighting vectors, whereas ***C*** ∈ ℝ^(*k*×^*^N^*^)^ contains time-varying activation coefficients; *k* is the number of extracted synergies and ***e*** denotes the reconstruction residual. Alternative formulations explicitly encode temporal structure, e.g., time-varying or spatiotemporal synergies that allow the patterns (or temporal kernels) to evolve over time, thus extending the basic factorization. By reducing dimensionality without discarding physiologically meaningful structure, synergies reduce the control burden while preserving task flexibility, improve noise tolerance, stabilize estimation across sessions, lower the number of learnable parameters, and facilitate real-time control. Embedding this modular representation within synergy-based neuromuscular controllers constrains estimation and control within a biologically plausible subspace.

Recent studies have increasingly integrated such synergy representations into neuromuscular controllers and musculoskeletal models, demonstrating their potential to reproduce coordinated muscle activation patterns in both simulated and experimental limb motor tasks. For example, Ruckert et al. employed time-varying synergies to accomplish sagittal-plane reaching in an open-loop scheme [11]. Chen et al. introduced a deep reinforcement learning (DRL) framework with memory and attention while constraining the policy to a predefined set of synergies derived from simulation, thus improving learning under imperfect observations but reducing physiological interpretability [12]. Han et al. addressed the challenge of learning human-like motor skills in embodied musculoskeletal models arising from high-dimensional and redundant muscle actuation [13]. Liu et al. proposed BioSynGrasp, a bio-inspired reinforcement learning framework to address the challenges of high-dimensional, coupled, and nonlinear control in anthropomorphic musculoskeletal robots during dexterous manipulation [14]. From an engineering perspective, instability in synergy structure can undermine the development of robust and reusable controllers for prosthetic and robotic applications. Although several studies have examined sources of inconsistency, important issues remain unresolved. These advances underscore the promise of synergy-informed neuromuscular control methods. However, they also show that synergy extraction is sensitive to methodological choices, raising concerns about robustness, consistency, and reliability.

Beyond proof-of-concept control, the broader literature in neuroscience, bio-inspired machine control, and engineering applications also demonstrates substantial promise for synergy-based intelligent systems. Notably, multiple studies report task dependence of synergies [15,16,17]. When performing the same or similar biomechanical tasks, the number and structure of muscle synergies identified across repeated movements would be expected to show reasonable consistency. However, large variability exists within and across studies for a given task, even for healthy individuals [18]. For tasks like postural control [19], gait [15,18,20], and upper-extremity movement [21] in healthy individuals, a low-dimensional representation consisting of approximately 4–6 synergies has been consistently identified [16,22]. However, the exact number and structure of synergies remain dependent on the task, population, anatomical region, recording set, and analytical pipeline. In addition, previous studies have shown substantial variability in the number of extracted muscle synergies in healthy individuals during walking, ranging from 2 to 6 [7,18,23]. Lencioni et al. [7] reported that, among 12 healthy subjects, 58% exhibited four muscle synergies and 42% exhibited three, while a similar distribution was observed in 17 persons with multiple sclerosis. Goudriaan et al. [23] did not directly determine the number of synergies but assessed motor-control complexity using the variance accounted for by one synergy (VAF). Moreover, variability in muscle synergies was observed during upper-extremity reaching tasks. The reliability and repeatability of muscle synergies in healthy individuals are essential in evaluating patients’ functional performance after rehabilitation [24]. In patient populations, the number of muscle synergies is typically in the range of 2 to 5 [18]. Thus, it is important to note that these muscle synergies might not be pathological. This overlap makes it difficult to distinguish disease-related changes from methodological variation when comparing healthy and clinical populations [25]. Therefore, synergy number alone may be insufficient for identifying functional impairment in clinical populations. Moreover, the robustness of synergy estimates during genuinely multi-DOF limb movements has been discussed far less thoroughly [26].

Despite growing interest, a persistent and critical challenge remains unresolved: the lack of methodological consistency and limited reliability across studies. Muscle synergies are typically extracted from sEMG signals by applying different factorization techniques. However, multiple methodological factors can influence the resulting synergy structure. In practice, synergy analyses vary widely in experimental design, acquisition protocols, electrode placement, and preprocessing (e.g., filtering and normalization) [24,27,28,29,30], as well as in decomposition algorithms and criteria for determining the number of synergies [31,32]. Comprehensive sEMG coverage is often infeasible, particularly for deep or small muscles, due to electrode and anatomical constraints; combined with heterogeneous setups and pipelines, this yields poor reproducibility and limits cross-study comparability [30]. Outcomes may be sensitive to methodological choices at every stage; variation in any step can alter the inferred synergy structure and complicate cross-study comparison. Physiologically, this complicates discrimination between true neural adaptations and methodological artifacts when comparing populations or tasks [18,23,24]. Pinpointing the most influential factors and quantifying how sensitive synergy estimates are to them is critical for reproducibility and for establishing muscle synergies as a dependable instrument in motor control.

In response to these issues, this review examines the methodological landscape of muscle synergy extraction within the framework of limb neuromechanical controllers. Whereas prior surveys by Turpin et al. [33] and Cheung et al. [34] synthesize broad evidence on muscle synergies, our focus is deliberately narrower: we identify and critically assess the specific methodological factors that determine the reliability and robustness of muscle-synergy analysis. Specifically, we analyze how choices across five dimensions shape decomposition outcomes: (1) experimental/acquisition design; (2) sEMG preprocessing and normalization; (3) factorization models and regularization, including time-varying and spatiotemporal variants; (4) model-order selection and stability; and (5) cross-trial/session/subject alignment and strategies for handling nonstationarity. We evaluate their impact on quantitative metrics (e.g., VAF and estimated module number) and qualitative properties. We conclude with recommendations that distinguish minimum reporting requirements from context-dependent analytical choices, with the aim of improving methodological consistency and physiological interpretability in muscle synergy analysis. Ultimately, clarifying how methodological choices influence synergy reliability can help bridge the gap between muscle-synergy analysis and its practical application in limb neuromechanical control, clinical rehabilitation, and engineering applications. Such methodological improvements may support the development of a more reproducible, interpretable, and broadly applicable synergy-based framework in related fields.

## 2. Terminology and Quantitative Criteria

To ensure terminological consistency throughout this review, the terms robustness, reliability, repeatability, reproducibility, stability, and similarity are distinguished according to their experimental context and intended interpretation, as shown in Table 1. Robustness refers to the extent to which extracted synergy results remain consistent under reasonable methodological variations. Reliability refers to the consistency of repeated measurements and is generally evaluated using repeated-measures assessments. Repeatability describes consistency under essentially identical experimental and analytical conditions, whereas reproducibility refers to the ability to obtain comparable results when relevant conditions differ, such as across sessions, laboratories, acquisition systems, or analytical settings. Stability refers primarily to the consistency of an extracted solution across repeated algorithmic runs, resampling, different initializations, or parameter variations, whereas similarity describes the quantitative resemblance between extracted synergy structures, as assessed by measures such as vector correlation, cosine similarity, or spatial similarity. Physiological validity refers to the extent to which the extracted mathematical modules reflect genuine neural control strategies, rather than patterns introduced by the decomposition algorithm or data-processing procedures.

These terms were retained when explicitly used in the original studies reviewed in this manuscript. However, their operational definitions, quantitative criteria, and thresholds vary across studies. Accordingly, reported evaluation metrics, cutoff values, and analytical procedures should be interpreted within the methodological context of each study. Rigorous statistical reporting, including 95% confidence intervals, specification of the intraclass correlation coefficient (ICC) model where applicable, and effect sizes (e.g., Cohen’s *d*), is recommended to support quantitative interpretation. Nevertheless, universally accepted absolute thresholds are not currently available across all muscle synergy extraction methods. Therefore, study-specific thresholds, equivalence bounds, and decision criteria should be predefined and clearly reported according to the analytical approach, study design, and biomechanical characteristics of the task.

## 3. Neural Basis of Muscle Synergies

**Figure 1:**
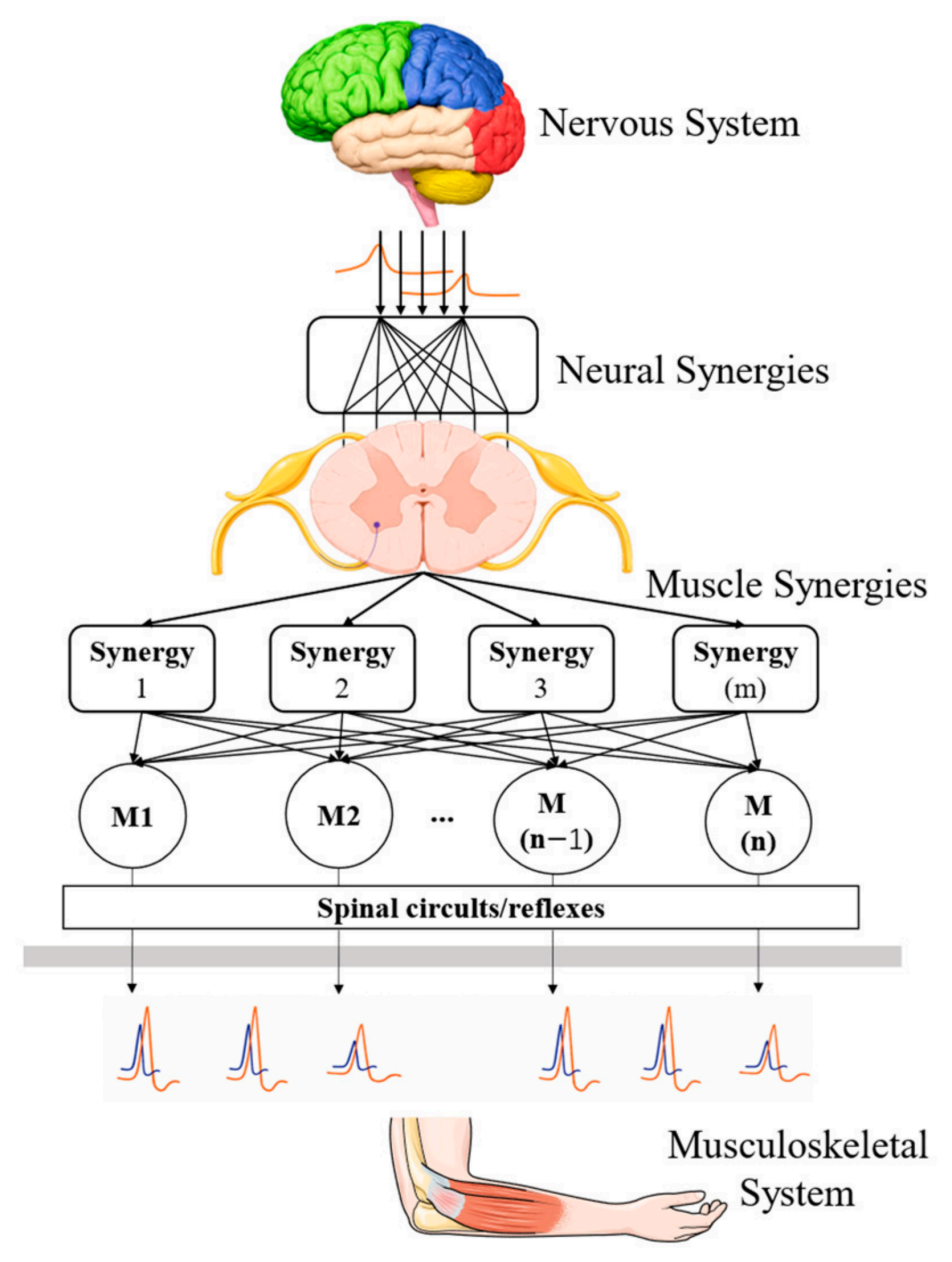
Conceptual framework of motor coordination based on neural and muscle synergies. Motor planning and descending commands generated by the CNS are integrated with sensory inputs and transmitted to spinal motor neuron pools, which ultimately regulate muscle activation. Motor coordination can be organized through a relatively small set of neural and muscle synergies, characterized by low-dimensional modular organization and flexible recruitment [53,54]. Individual motor tasks are generated by recruiting multiple synergies (Synergy 1, Synergy 2, …, Synergy *m*) with task-dependent weighting coefficients. Each muscle synergy represents the coordinated activation of multiple muscles (M1, M2, …, M*n*), and the resulting sEMG signals reflect the superposition of these recruited muscle activation patterns. Muscle–tendon units subsequently transform neural activation into muscle forces, producing coordinated multi-joint actions and interactions with the external environment. Through this hierarchical and modular process, a limited repertoire of synergies can generate a broad range of voluntary movements and motor behaviors.

The execution of human voluntary movements requires coordinated interactions between the nervous and musculoskeletal systems [42,43], as shown in Figure 1. Neural processing encompasses several stages at different levels of the nervous system (i.e., cortical and spinal) and the resultant synaptic inputs are transmitted to spinal motor neuron pools [44]. Then, the spinal motor neurons are recruited to activate the corresponding muscles to produce coordinated motions. One major challenge is how the CNS deals with this highly redundant and nonlinear system, which includes numerous degrees of freedom and motor units, to generate flexible and adaptive movements [45]. A long-standing hypothesis in motor control is that the CNS handles the high dimensionality of muscle activations with a hierarchical architecture composed of discrete basic motor modules [6]. From the perspective of motor control of complex systems, a hierarchical decomposition with modules, or building blocks, is employed as the most effective strategy to perform tasks [46]. Over the past century, several primitive hypotheses have been proposed about the modular organization underlying movement generation, i.e., the half-center model [47], neuromotor synergies [48], and unit burst generators [49]. More importantly, some studies have demonstrated that the output of a module can be characterized as a force field generated by discrete groups of coordinately activated muscles, commonly described as muscle modules or synergies [8,9]. These basic organizational principles can be used to interpret the rhythmic behaviors generated at the spinal cord level.

Building upon these early theoretical foundations, researchers have increasingly turned to animal models to explore the modular basis of motor control. Studies in animal models, including turtles [50], frogs [8,10], rats, and cats [16], have provided substantial evidence. With different approaches such as micro-stimulation of the spinal cord [10], N-methyl-D-aspartate iontophoresis [51], and examination of natural behaviors in intact and deafferented animals, evidence supports the concept that modular organizations can generate motor behaviors. In other words, these behaviors were generated by simultaneously recruiting and coordinating sets of muscles, thus supporting the hypothesis of modular organizations in motor control. The CNS may utilize the modular organization shown in Figure 1 to generate motor commands. By doing so, it can substantially reduce computational load and help to address the redundancy inherent in motor control [7,8,10]. Evidence has also emerged suggesting that similar organizational principles may contribute to the generation of voluntary movement in humans. A previous study suggested that muscle synergies were structured in the brainstem or spinal cord [52]. Furthermore, the organization of muscle synergies develops and adapts progressively across different developmental stages, from early childhood to adulthood.

## 4. Muscle Synergy Extraction Pipelines Using EMG

**Figure 2:**
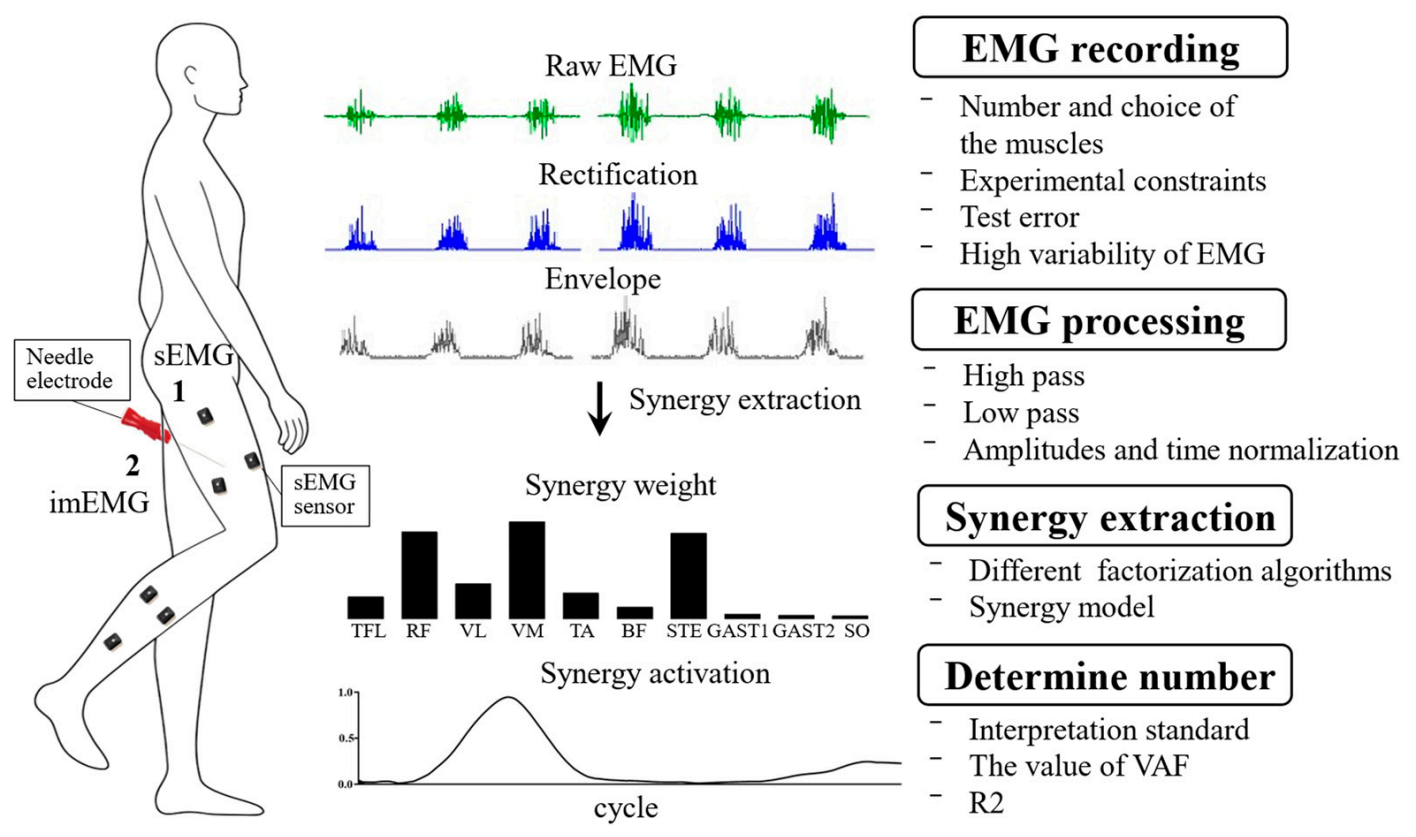
Steps of muscle synergies analysis.

Muscle synergy analysis infers control strategies from the variability and coordination of multi-muscle sEMG recorded during movement. A typical pipeline comprises (i) acquiring sEMG from multiple muscles during one or more behaviors; (ii) preprocessing to attenuate environmental noise, baseline drift, and electrode or skin motion artifacts [30,55,56]; (iii) factorizing the processed sEMG to estimate the synergy structure *W* and activation coefficients *C*; (iv) selecting an appropriate number of synergies; and (v) evaluating the extracted synergies and their downstream performance [57]. Each step can affect robustness and reliability—through experimental design, the number and selection of muscles [32,58], preprocessing choices [29], factorization techniques [59,60], and model-order criteria, among others. Next, this review analyzes these methodological dimensions within that pipeline, as shown in Figure 2, and the key methodological factors affecting synergy extraction are summarized in Table 2.

### 4.1. Decomposition via Computational Methods

The concept of muscle synergies has been formalized through various mathematical frameworks aimed at decomposing complex EMG signals into lower-dimensional representations of motor control. Depending on how temporal and spatial structures are represented, synergy models are typically grouped into three primary types that characterize coordination at different levels: spatial, temporal, and spatiotemporal dimensions [61]. The first category comprises synergies identified as fixed vectors, which are often referred to as synchronous synergies (i.e., time-invariant, or spatial muscle synergies). The synchronous synergies are built from synergistic groups of muscles activated with a fixed time course [62]. In contrast, the temporal synergy model assumes that activations of the synergies are sequenced similarly across movement cycles or tasks, and the weightings can vary in time. It represents invariant muscle activation waveforms across muscles and conditions [63,64]. Another proposed formulation is spatiotemporal muscle synergy, in which synergy vectors are both shifted in amplitude and scaled in time [65,66]. Collectively, these formulations provide complementary perspectives on how the CNS may organize muscle activations.

A major source of inconsistency across studies is that synergy estimates depend strongly on the chosen model class: the inferred number and structure of synergies, their stability, and cross-study comparability can all change with spatial vs. temporal vs. spatiotemporal assumptions. Spatial modules are often hypothesized to reflect spinal/corticospinal primitives, whereas time-varying patterns may relate to higher-level cortical–cerebellar coordination. Consequently, careful model selection and transparent reporting of assumptions are essential for interpretable, comparable synergy analysis.

### 4.2. Experimental Design and EMG Signal Acquisition

The acquisition of high-quality EMG data is the foundation for reliable synergy extraction. Experimental design decisions, including the number of muscles recorded, electrode configuration, task protocol, and inter-individual variability, strongly influence both the signal quality and the stability of subsequent factorization [67,68]. For upper-limb studies, EMG signals are typically recorded from multiple muscles spanning the shoulder, elbow, and wrist joints to capture inter-joint coordination. However, anatomical constraints, sensor placement limitations, and task-specific variability often prevent comprehensive muscle coverage. For instance, deep or small muscles (e.g., iliopsoas) are difficult to measure using surface electrodes but may still contribute substantially to motor coordination [69].

Currently, no standardized guideline defines how many or which muscles should be included in EMG recordings for synergy analysis [32]. In practice, upper-limb studies typically record between 8 and 19 muscles [21,22,70], while gait and lower-limb analyses typically include 8–16 muscles [16,25]. Muscle selection often represents a balance between anatomical coverage, task relevance, and signal quality. To ensure comparability across studies, it is critical to document muscle selection, electrode placement, and other relevant acquisition parameters. Other factors affecting EMG acquisition include (1) electrode placement and inter-electrode distance, which determine selectivity and cross-talk sensitivity; (2) task design, such as movement direction, load, or speed, influencing activation timing; (3) participant-related factors, including muscle morphology, skin impedance, fatigue, and learning effects; (4) instrumentation parameters, such as sampling frequency, filtering (20–450 Hz), and differential amplification to improve SNR.

Given the complexity of the musculoskeletal system, which comprises more than 600 muscles [71,72], it is not feasible to record the activation of all muscles contributing to complex upper-limb tasks. Therefore, EMG acquisition should aim for representative rather than complete muscle coverage, ensuring that the recorded muscle set sufficiently captures the functional synergies relevant to the task. Clear reporting of experimental configurations, electrode placement, and participant-specific adjustments is essential for reproducibility and facilitating cross-study comparisons.

### 4.3. Preprocessing of EMG Signals

sEMG provides physiological information on muscle activation that can be combined with computational methods to investigate neuromuscular coordination [68]. The characteristics of sEMG signals, especially the amplitude, temporal and frequency features, might be affected by the skin condition, electrode placement, movement artifacts, and other noise sources that are inherent and inevitable. For example, cross-talk among sEMG channels would be mistakenly treated as synergistic activity and influence muscle synergy structure [73,74,75]. In addition, environmental and processing factors might affect the estimation of linear envelopes during sEMG measurement. Hence, a high-quality signal is an important foundation for sEMG applications [76]. To ensure the quality of the signal, the acquisition procedures and recommendations proposed by the Surface Electromyography for the Non-Invasive Assessment of Muscles project (SENIAM) were generally followed [77,78]. The objective of sEMG preprocessing is to acquire an accurate description of muscle activation for factorization across various tasks.

To ensure high-quality signals for muscle synergy extraction, sEMG preprocessing must reliably separate physiological muscle activation from environmental noise and movement artifacts. Following standard SENIAM recommendations, the processing pipeline typically follows a strict sequential structure:

(1) Raw-EMG Filtering: Raw signals are first processed using a band-pass filter to attenuate motion artifacts and high-frequency noise. High-pass (HP) cutoffs typically range from 5 to 20 Hz [79] (sometimes up to 50 Hz [30,80,81] in the presence of severe artifacts), with low-pass (LP) cutoffs set at approximately 400–500 Hz. If low-frequency drift persists, baseline correction is performed prior to further processing; in cases of severe artifacts, channel rejection or segment masking is preferred over overly aggressive filtering.
(2) Rectification: The filtered raw signal is then subjected to full-wave rectification.
(3) Envelope Generation: A separate LP filter is subsequently applied to the rectified signal to extract the linear envelope. Cutoff frequencies at this stage typically range from 2 to 15 Hz [18,20,80,82,83]. Because the reviewed literature does not support a single setting, this specific cutoff must be explicitly tailored to the task dynamics and analytical objectives, balancing necessary temporal resolution with noise suppression [84,85].
(4) Temporal Normalization: To facilitate comparison across trials, sessions, and subjects, the envelope profiles are often aligned to specific kinematic events and time-normalized (e.g., to 100% of a movement cycle).
(5) Amplitude Normalization: This final step is required to mitigate inter-subject and inter-session variability. Common scaling options include the maximum amplitude of the signal itself (SELF) [18,21,30,86], maximal voluntary contraction (MVC) [87,88], median trial maximums [21], or unit variance scaling [16,89,90]. Unit variance normalization is frequently preferred as it prevents high-variance muscles from dominating the decomposition, yielding more stable synergy patterns [16,91]. However, because comparative studies show varying results and no universal standard exists, the normalization method remains highly context-dependent [92].

Preprocessing choices at every stage can alter the spectral and temporal characteristics of the data and thereby influence synergy weights, activation profiles, and the estimated number of synergies. Transparent reporting of the full processing sequence and its consistent application are essential for reproducible synergy extraction [93].

### 4.4. Techniques for Muscle Synergy Extraction

Common dimensionality-reduction methods for extracting muscle synergies from preprocessed sEMG include independent component analysis (ICA), nonnegative matrix factorization (NMF) [94], principal component analysis (PCA) [95], and factor analysis (FA) [70]. Depending on the assumptions and implementation of each method, these matrix factorization approaches construct lower-dimensional representations of muscle activation patterns according to different statistical or optimization objectives [60].

For instance, PCA constrains the columns of ***W*** to be orthonormal and the rows of ***C*** to be orthogonal to each other. The PCA orthogonal assumption permits negative values in the synergy subspace [34]. PCA identifies orthogonal components that sequentially maximize the variance explained in the data [95]. Unlike PCA, ICA assumes that muscle synergies represent independent sources of information [96]. ICA is designed to extract statistically independent components, typically by first whitening the data to remove second-order correlations and then rotating the whitened data to maximize statistical independence or non-Gaussianity among the extracted components. Thus, whitening alone does not establish independence but provides a preprocessing step for subsequent independent-component estimation.

Similarly to PCA, FA employs eigenvalue decomposition to obtain eigenvectors of the covariance matrix [97] and seeks the smallest set of synergies to explain the common variance of a set of muscles. It is noteworthy that the number of degrees of freedom can affect the number of basis vectors in the muscle synergy model when assessed using FA. NMF is a machine-learning method that decomposes a high-dimensional nonnegative matrix into two nonnegative matrices [94]. A distinctive feature of NMF is its non-negativity constraint, which is compatible with the nonnegative nature of muscle activation amplitudes and facilitates an additive representation of muscle coordination patterns. Because the NMF optimization problem is non-convex, factor initialization can affect convergence and the resulting solution. Traditional implementations often use random nonnegative initializations, and different initializations may converge to different local optima. Accordingly, multiple runs with different initializations are commonly performed, and candidate solutions should be evaluated using predefined reconstruction-error, stability, or combined criteria. Singular value decomposition (SVD)-based initialization and sparse NMF constraints may improve convergence behavior or promote sparsity, depending on the analytical objective [98]. In addition to conventional NMF, alternative optimization and factorization approaches have been explored, including improved optimization strategies [61], multivariate curve resolution–alternating least squares (MCR-ALS) [35], and mixed matrix factorization (MMF) [99].

Beyond conventional linear matrix decomposition methods, several advanced models and computational frameworks have been developed to address specific limitations of muscle synergy analysis. These approaches should not be considered universally superior to linear methods. Their suitability depends on the balance between identifiability, physiological plausibility, and interpretability, robustness to noise and methodological perturbations, and computational burden.

(1) Probabilistic and Bayesian frameworks incorporate an explicit noise model and can quantify uncertainty in model parameters and, in some formulations, in the number of synergies. This feature may improve the interpretation of uncertain or noisy data and provide a principled basis for model comparison. However, the identifiability of the extracted synergies depends strongly on the assumed likelihood, prior distributions, model constraints, and available data quantity. Posterior inference and model comparison may also require substantially greater computational resources than conventional NMF.
(2) Tensor-based decomposition extends matrix factorization to multidimensional data arrays and can preserve task-, trial-, and subject-specific structures that may be lost when the data are collapsed into a two-dimensional matrix [100]. This may better preserve the representation of spatiotemporal and cross-condition relationships. However, tensor methods require careful alignment of data dimensions and appropriate selection of tensor rank. Their identifiability and robustness depend on uniqueness conditions, preprocessing quality, missing-data structure, and imposed constraints. The additional data modes and parameters also increase computational complexity.
(3) Nonlinear and deep latent-variable models, including autoencoder-based approaches, can represent nonlinear relationships in EMG data that may not be adequately captured by linear decomposition methods [41]. They may improve reconstruction or prediction under particular datasets and noise conditions, but high reconstruction performance should not be equated with physiological validity. Their robustness to distribution shifts, changes in initialization, and experimental perturbations remains data-dependent. These models generally require more training data and computational resources, and their latent variables may be difficult to identify uniquely or interpret physiologically.
(4) Time-varying synergy models explicitly represent temporal changes in muscle coordination and are therefore particularly relevant to dynamic motor behaviors, although this additional flexibility increases model complexity [8,70]. By allowing synergy weights or activation profiles to evolve over time, these models can describe changes in muscle coordination that are not represented by time-invariant linear models. The additional temporal parameters may also increase computational burden. Because time-varying synergy weights and activation coefficients may be partially confounded, their identifiability requires appropriate constraints, regularization, or independent validation. Without such safeguards, the increased flexibility may reduce solution stability and increase the risk of overfitting.

Overall, these methods represent high-dimensional EMG signals using lower-dimensional or structured latent components, but their differing assumptions and optimization objectives necessitate evaluation across multiple dimensions, including reconstruction quality, stability, physiological plausibility, interpretability, robustness to noise and methodological perturbation, and computational cost. Importantly, a rigorous quantitative comparison across these methods would require them to be evaluated on the same dataset using identical or standardized preprocessing procedures, experimental conditions, parameter settings, and evaluation criteria. Otherwise, differences in data characteristics, signal quality, channel number, task design, preprocessing, and implementation may confound direct comparisons.

Moreover, there is currently no universally accepted set of quantitative criteria or fixed thresholds for evaluating identifiability, robustness, computational burden, physiological interpretability, and intended use across all methods. Therefore, these dimensions should be considered collectively rather than treated as absolute criteria or used to establish a fixed ranking among NMF, PCA, ICA, FA, MCR-ALS, nonlinear models, and deep-learning-based approaches. Method selection should instead be guided by specific research objectives, such as dimensionality reduction, source separation, physiological interpretation, real-time implementation, or predictive performance.

### 4.5. Determining Synergy Number

VAF is a common metric used to evaluate how well a muscle synergy model reconstructs the original EMG signals. VAF is commonly assessed at two levels: Total VAF and Local VAF. Total VAF, which evaluates the overall reconstruction accuracy across all recorded muscles and time points, is calculated according to a previous study [101] as

(2) $$\begin{array}{c} \mathrm{Total VAF}=\left[1-\frac{\sum_{i=1}^{m}\sum_{j=1}^{N}{\left(e_{i,j}\right)}^{2}}{\sum_{i=1}^{m}\sum_{j=1}^{N}{\left(X_{i,j}\right)}^{2}}\right]\times 100\% \end{array}$$

The local VAF was calculated separately for each muscle based on Hug et al. [102]:

(3) $$\begin{array}{c} \mathrm{Local VAF}\left[m\right]=\left[1-\frac{\sum_{j=1}^{N}{\left(e_{m,j}\right)}^{2}}{\sum_{j=1}^{N}{\left(X_{m,j}\right)}^{2}}\right]\times 100\% \end{array}$$

Here, *X_i_*_,*j*_ denotes the original EMG value of the *i*-th muscle at the *j*-th sampled time point, whereas *e_i_*_,*j*_ denotes the corresponding reconstruction residual. The index *i* = 1,…, *m* refers to the recorded muscles, where *m* is the total number of muscles, and *j* = 1,…,*N* refers to the sampled time points, where *N* is the total number of sampled time points. *e_m_*_,*j*_ denotes the *m*-th muscle at the *j*-th sampled time point reconstruction residual, *X_m_*_,*j*_ denotes the original EMG value of the *m*-th muscle at the *j*-th sampled time point. Total VAF evaluates the global mathematical performance of the synergy model across the entire dataset. However, a high Total VAF can sometimes mask poorly reconstructed individual muscles. Therefore, Local VAF may be evaluated concurrently to ensure no functionally critical muscle is discarded. The number of synergies is determined by sequentially increasing the model order and selecting the smallest number of synergies for which the Total VAF and all Local VAF values satisfy the predefined acceptance criteria. The optimal model order can then be defined as the smallest number of synergies for which the predefined global and local reconstruction criteria are satisfied.

High VAF values indicate that the synergy model provides an accurate reconstruction of the recorded EMG signals. Conversely, low VAF values indicate substantial unexplained variability in EMG data. Under this assumption, the number of muscle synergies is commonly determined using several VAF-based approaches, including the fixed threshold method [103], best linear fit method [104], knee point method [21], and the “elbow” method [105]. In a fixed-threshold approach, the selected number is the smallest number of synergies that reaches a pre-specified total VAF criterion. Torres-Oviedo et al. [16] proposed a fixed threshold method using a VAF threshold of 90% to determine the number of muscle synergies for extraction. The threshold should be reported explicitly because values used across studies differ. Cheung et al. [104] presented the best linear fit method, which progressively increases the number of synergies and fits the residual portion of the VAF curve until the mean squared error (MSE) falls below the predefined criterion. The point at which the VAF curve plateaus to a straight line is then selected as the number of muscle synergies. The knee point method is assessed by computing the changes in the slope or curvature of every three adjacent points in the VAF-versus-number-of-synergies curve. The number of muscle synergies is identified at the point beyond which the incremental improvement in VAF becomes relatively small [21,103]. Finally, Tresch et al. [105] suggested selecting the point at which the change in the slope of the curve was greatest. Such an “elbow” was interpreted as the point separating “structured” and noise-dependent variability. It can be used to define the minimum number of synergies that capture the task-related features. Besides the VAF metric, other criteria have also been proposed to determine the number of synergies. The reported VAF cutoff thresholds commonly range from 80% to 95% [106,107]. Accordingly, the appropriate threshold should be interpreted in relation to the task, dataset, noise level, population, and validation objective. Inconsistent thresholds make cross-study comparisons difficult, and synergy-number estimates should therefore be reported together with the chosen criterion and appropriate sensitivity analyses [17].

Furthermore, interpretation of these thresholds requires a clear distinction between reconstruction quality, solution stability, and physiological validity. VAF quantifies reconstruction quality by measuring how well the extracted matrices reproduce the original EMG signal. Although a VAF exceeding 90% is often considered evidence of satisfactory decomposition, it does not establish that the extracted modules represent a physiologically correct solution. The residual EMG not explained by the model may contain functionally relevant, task-specific neural information rather than noise alone. Thus, a high VAF supports the mathematical adequacy of approximation but cannot, by itself, demonstrate that the extracted modules reflect genuine neural drives. The selected number of synergies should therefore also be evaluated for solution stability by examining consistency across repeated algorithmic initializations and supported by evidence of physiological validity, such as the ability to predict biomechanical outputs.

In general, these criteria aim to determine the number of synergy vectors required to represent the structural variability within a dataset [66,108]. Additionally, criteria based on the linear fit of the coefficient of determination (R^2^) and other criteria, such as Akaike Information Criterion (AIC), Minimum Description Length (MDL) or Bayesian Information Criterion (BIC), have also been proposed to determine the number of synergies [108,109,110]. In practice, models with different numbers of synergies are fitted separately, and the model yielding the lowest criterion value is selected. AIC balances reconstruction goodness-of-fit against model complexity by imposing a penalty proportional to the number of estimated parameters. MDL selects the model that minimizes the total description length of both the model and the residual data; therefore, an additional synergy is retained only when the improvement in data description exceeds the extra complexity it introduces. BIC uses a similar likelihood–complexity trade-off but applies a stronger penalty that increases with sample size [111]. However, their estimates depend on the assumed noise distribution, the definition of the effective sample size, and the parameter-counting scheme. This issue is particularly relevant for EMG data, in which temporal autocorrelation means that successive samples may not be statistically independent [112]. To address the time-series autocorrelation inherent in continuous physiological signals, the effective sample size ($\begin{array}{c} N_{\mathrm{eff}} \end{array}$) must be adjusted rather than using the raw number of time points ($\begin{array}{c} N \end{array}$). For LP-filtered EMG data, $\begin{array}{c} N_{\mathrm{eff}} \end{array}$ can be statistically estimated using the lag-1 temporal autocorrelation coefficient ($\begin{array}{c} \rho \end{array}$) via the approximation $\begin{array}{c} N_{\mathrm{eff}}\approx N\;\frac{1-\rho }{1+\rho } \end{array}$. This mandatory adjustment ensures the correct statistical mechanics are applied to the penalty terms in AIC and BIC, preventing the over-penalization of model complexity. Consequently, using the total number of sampled time points may overestimate the effective sample size and affect model-order selection [113]. Accordingly, the effective sample size should account for temporal dependence and may be estimated from the autocorrelation structure.

Importantly, these model-order criteria are not restricted to conventional NMF-based analysis and may also be incorporated, with appropriate formulation, into more advanced probabilistic or Bayesian frameworks. In such approaches, uncertainty in model parameters and, in some cases, model order can be represented explicitly, although identifiability may remain dependent on prior assumptions, model constraints, and data quantity. However, the number of muscle synergies may be overestimated in the presence of signal noise [105]. Although these methods provide quantitative approaches for estimating synergy number, no universally accepted standard currently exists, and even apparently appropriate criteria may produce different results across studies [114].

## 5. Analysis of How Critical Factors Impact the Robustness of Muscle Synergies

This narrative review employed a structured literature search to identify and synthesize evidence on methodological factors influencing the robustness, reliability, and interpretability of muscle-synergy extraction within limb neuromechanical controllers. Relevant literature published from January 2000 to July 2026 was identified through PubMed, ScienceDirect, and Google Scholar. The search strategy combined terms related to muscle-synergy analysis and methodological evaluation: (“muscle synergy” OR “muscle synergies”) AND (“muscle coordination” OR “motor control” OR “motor primitive” OR “motor module” OR “electromyography” OR “sEMG”) AND (“methodology” OR “robustness” OR “reliability” OR “repeatability” OR “reproducibility” OR “factorization”). For ScienceDirect, the search string was adapted to comply with the database limit on Boolean connectors: (“muscle synergy” OR “muscle synergies”) AND (“electromyography” OR “sEMG” OR “motor control”) AND (“methodology” OR “reliability” OR “factorization”). The search syntax was adapted as necessary for each database. Reference lists of relevant original studies, methodological reviews, and key theoretical articles were also manually screened to identify additional publications.

Because this review was designed as a narrative rather than a systematic review, the literature search aimed to identify representative studies addressing major methodological approaches and key technical issues relevant to muscle synergy analysis. Studies were considered eligible if they met the following criteria: (1) they were full-text, peer-reviewed journal articles or conference papers published in English and provided sufficient methodological and results information; (2) they investigated muscle synergy extraction or analytical factors directly relevant to synergy estimation, including EMG acquisition and processing, matrix factorization algorithms, determination of the number of synergies, and experimental conditions; (3) they provided methodological information related to experimental design, EMG acquisition, preprocessing, factorization, model-order determination, robustness, reliability, or validation; and (4) they introduced, applied, or evaluated an approach of methodological relevance to muscle synergy analysis. Studies were excluded if they were not directly related to muscle synergy analysis, provided insufficient methodological information, or were available only as abstracts or non-peer-reviewed reports.

The electronic searches identified 513 records from PubMed, 890 from ScienceDirect, and 12,200 from Google Scholar. From the Google Scholar search results, the first 400 records ranked by relevance were retained for further screening. Records retrieved from the three databases were then combined, and additional studies were identified through manual screening of the reference lists of eligible articles and relevant reviews. After duplicate removal, the titles and abstracts of the remaining records were screened to identify studies explicitly relevant to muscle synergy methodology. Full-text articles were subsequently assessed according to the predefined eligibility criteria, and the most relevant studies were included in the review. This review was designed as a narrative review, with the literature search aimed at identifying representative studies addressing the major methodological approaches and key technical issues in muscle synergy analysis. Although the initial retrieval counts were retained and the above screening procedures were performed, a Preferred Reporting Items for Systematic Reviews and Meta-Analyses (PRISMA)-style screening log was not prospectively maintained. The studies included in this review were selected on the basis of their relevance and their ability to provide representative methodological evidence across the major domains addressed.

### 5.1. Different Experimental Constraints and Subjects

According to the reviewed studies, the robustness of muscle synergy structure appears to be context-dependent. Several studies reported relatively preserved synergy organization across controlled changes in biomechanical or sensorimotor conditions, as shown in Table 3. Torres-Oviedo et al. [16] identified five functional synergies that remained robust across multiple postural conditions in a feline model, whereas Roh et al. [22] reported relatively consistent synergy composition across spatial, load, and arm-position protocols in healthy participants. Gentner et al. [115] found that synergy structure was preserved during visuomotor adaptation. Together, these findings support a degree of robustness under controlled experimental conditions. However, their direct comparability is limited by substantial differences in species, task type, and experimental design, and therefore they do not establish that muscle synergy structure is invariant across motor behaviors.

Other studies highlight important limits to this apparent robustness. Augenstein et al. [28] showed that insufficient sampling of reaching directions altered the extracted synergy composition, indicating that the apparent validity of synergy structure depends partly on the breadth of the task space sampled. Geng et al. [116] found modest adjustments in forearm synergy composition across changes in force level and arm position, whereas Munoz-Martel et al. [19] reported recruitment of an additional complementary synergy under unstable balance conditions. These findings are not necessarily contradictory to the studies reporting preserved synergy structure; rather, they indicate that synergy organization may remain relatively stable under moderate variations in task conditions but may change when task demands, perturbation magnitude, or task-space coverage are altered more substantially. Accordingly, robustness should be interpreted relative to the range of experimental conditions actually tested rather than treated as an intrinsic and universal property of the extracted modules.

Subject- and environment-related variability represent additional sources of heterogeneity. Scano et al. [27] reported that a reduced set of muscle modules remained similar and repeatable across subjects and sessions despite substantial variability in upper-limb movements. Zhao et al. [36] evaluated intra-subject and inter-individual variability by comparing cosine similarity values with a data-driven random similarity reference. The random similarity was calculated as the mean similarity of paired synergy vectors pooled across all subjects and repetitions. Similarities were considered higher than the random reference when the statistical comparison was significant at *p* < 0.05. Therefore, the random matching level was not a fixed universal threshold, but rather an empirical reference derived from the specific dataset analyzed.

Evidence for repeatability is more heterogeneous and appears to depend strongly on the specific synergy outcome and acquisition procedure. Taborri et al. [37] reported that synergy number was not repeatable within or between subjects and identified intrinsic sEMG variability and electrode repositioning as important sources of variation. Pale et al. [117] similarly found lower spatial similarity between sessions than within sessions and showed that electrode repositioning altered synergy similarity. In contrast, other studies reported high repeatability of synergy vectors and activation coefficients under controlled conditions [38,39], while Shuman et al. [40] found that synergy complexity and structure were repeatable across days when EMG signals were averaged over multiple gait cycles in both healthy participants and individuals with cerebral palsy. These apparently divergent findings may therefore arise partly because different studies evaluated different properties of synergy solutions, such as synergy number, spatial vectors, and temporal coefficients, and used different populations, acquisition procedures, and averaging strategies.

Overall, the reviewed evidence supports a context-dependent interpretation of muscle synergy robustness and repeatability. Synergy composition can remain relatively stable under controlled or moderately varying conditions, but this stability becomes less consistent when task sampling is insufficient or when subject-, session-, and acquisition-related variability increases. Evidence appears more consistent for the repeatability of synergy vectors and temporal activation patterns under standardized or averaged recording conditions than for the repeatability of synergy number or cross-session similarity. Importantly, the reviewed studies differ substantially in population, task design, acquisition protocol, and validation metric; therefore, apparent agreement or disagreement across studies should not be interpreted without considering these methodological differences. The available evidence supports conditional stability of extracted synergies, rather than universal invariance across tasks, populations, and recording conditions.

### 5.2. Muscle Selection and EMG Acquisition

EMG acquisition for muscle synergy analysis involves an inherent trade-off between comprehensive anatomical coverage and experimental feasibility. sEMG has been widely used in clinical, robotic, and sports applications [67], but signal quality and interpretability can be affected by experimental constraints, inter-individual variability, testing duration, and the number and configuration of recording channels [68], including electrode spacing and inter-subject differences in muscle activation [118]. Because the human musculoskeletal system contains more than 600 skeletal muscles [71,72], recording all muscles involved in a motor task is generally impractical, and no universal standard currently defines either the number or identity of muscles that should be recorded [32]. Previous upper-limb studies have typically recorded approximately 8–19 muscles [21,22,70], whereas gait studies have commonly included 8–16 lower-limb muscles [16,25], with relatively few studies able to sample all task-relevant muscles [119]. This limitation is particularly important for small or deep muscles that are inaccessible to surface electrodes [69]. Musculoskeletal simulations further suggest that unrecorded muscles may still contribute substantially to task-specific control; for example, Neptune et al. [15] identified an iliopsoas-related module contributing to leg advancement, while additional modeling work indicated that omitted muscles may participate in balance and non-sagittal-plane control [120]. Thus, incomplete muscle sampling may limit the physiological interpretation of extracted synergies [121], and both the number and identity of recorded muscles should be considered when interpreting synergy outcomes [31,32].

As shown in Table 4, comparative studies provide convergent evidence that reducing or altering the recorded muscle set does not necessarily eliminate the dominant synergy structure but may affect specific analytical outcomes. Steele et al. [32] found that synergy similarity remained above 0.80 when more than 10 muscles were retained, although reducing the muscle set increased the risk of VAF overestimation. Similarly, Saito et al. [58] showed that inclusion of deeper lower-limb muscles preserved three of four major synergy structures while altering one. Brambilla et al. [122] further reported that a 32-muscle configuration required one additional synergy compared with a 12-muscle configuration to achieve comparable reconstruction performance, despite core synergy similarity remaining above 0.97. Taken together, these findings indicate that reduced muscle sets may preserve dominant modules while still modifying reconstruction quality, estimated synergy number, or task-specific muscle contributions. Importantly, the magnitude of these effects differed across tasks and muscle configurations, so numerical findings from individual studies should not be interpreted as universal minimum muscle requirements.

The suitability of a reduced muscle set also depends on the intended analytical objective and the method used for muscle selection. Chen et al. [123] proposed the determination of optimal muscle synergies from estimated muscle activations (DOMSn-EMAs) framework to achieve more reliable and physiologically meaningful muscle synergies. It combines high-fidelity musculoskeletal modeling with a modified computed muscle control algorithm to generate high-density estimated muscle activations, then applies sparse NMF to extract synergy patterns and uses a composite reliability index to select the optimal number of synergies. In myoelectric control, Camardella et al. [124] evaluated all possible combinations of 15 candidate muscles. The analysis identified an eight-muscle global optimal set and a six-muscle subject-specific set, corresponding to reductions of seven and nine muscles from the 15-muscle candidate pool, respectively. The reduced sets produced synergy similarity values ranging from 0.74 to 0.78, while their force-estimation performance was not significantly different from that of the full set. Guez et al. [125] compared variance-based PCA with model-based recursive feature elimination for muscle selection. They found that recursive feature elimination (RFE)-chosen muscles exhibited strong correlation with subjects’ synergy structures, whereas PCA tended to favor muscles with high variability rather than those representative of synergies, underscoring the critical role of physiological information in effective channel selection.

Musculoskeletal and humanoid optimization studies provide complementary but more indirect evidence. Genetic-algorithm and convex-hull approaches have shown that redundancy can be reduced while preserving task performance in musculoskeletal or robotic systems [126,127,128]. However, these findings do not directly establish the optimal number of muscles required for experimental human sEMG-based synergy analysis. Overall, the available evidence therefore supports task-relevant and anatomically representative muscle selection rather than a fixed universal number of channels. Reduced muscle sets may be appropriate in some contexts, but their validity should be evaluated against the intended outcome, including reconstruction quality, synergy similarity, model order, and task-specific muscle representation. Musculoskeletal modeling may provide complementary information for muscles that cannot be directly recorded but should not be regarded as direct evidence defining an optimal experimental muscle set.

**Table 4:** Studies investigated muscle synergies on sEMG acquisition and preprocessing.

Study	Study Category (Sample Size N = Subject Number)	Tasks	Methods				Outcomes
			Recorded Muscles	EMG Preprocessing	Synergy Extraction	Analysis Criteria	
Banks [24]	Clinical populations (N = 10)	Walking.	TA, MG, SO, RF, VL, BF, ST, GM.	BP: 20–500 Hz, Demeaned, Rectified, LP: 7 Hz.	NMF	VAF > 90%	EMG normalization had a great effect on stride-to-stride variability of the synergy vectors. The number of synergies impacted the variability of both synergy vectors and synergy activations.
Santuz [29]	Human participants (N = 20)	Walking and running.	LD, DELT1, DELT3, TRAPS, BB, TRI Long, ES, SPL, RA, EO, ST, BF, VL, VM, RF, TFL, AL, GM, GLU, TA, MG, GL, SO, PL.	HP: 20, 50, 250 Hz, Rectified, LP: 5, 10, 20 Hz, Normalized.	NMF, Gaussian NMF, inverse-Gaussian NMF.	R^2^ > 0.95 AIC	Compared with the GNMF method, the IGNMF had superiority in reconstructing original EMG signals through one less synergy on average. The fourth order, 50 Hz and LP 20 Hz could be the most appropriate EMG smoothing combination.
Shuman [30]	Clinical populations (N = 186)	Gait analysis.	RF, MH, LH, MG, TA.	HP: 40 Hz, Rectified, LP: 4–40 Hz, Normalized.	NMF	VAF	The choice of LP filter and unit variance normalization had a larger impact on total VAF than synergy weights and activation; Z-score normalization may improve comparability across studies.
Steele [32]	Simulations	Reaching.	DELT1, DELT2, DELT3, SUPSP, INFRA, SUBSC, TMIN, TMAJ, PT, PECM1, PECM2, PECM3, LAT1, LAT2, LAT3, CORB, TRI Long, TRI Lat, BIC Long, ANC, SUP, BIC Long, BIC Short, BRA, BRD, ECRL, ECRB, ECU, FCR, FCU.	Not reported	NMF	VAF	Using fewer muscles in an experimental analysis would increase the risk of over-estimating the VAF. Similarity remained over 0.8 with more than 10 muscles.
Saito [58]	Human participants (N = 12)	Walked on a motorized treadmill.	VL, VM, RF, BF, SM, AM, AL, MG, TA.	BP: 20–450 Hz.	NMF	VAF > 90%	Muscle synergies extracted from a muscle set that included deep muscles appeared to be relatively consistent between level and uphill treadmill walking in healthy participants.
Brambilla [122]	Simulations	Reaching.	DELT1, DELT2, DELT3, SUPSP, INFRA, SUBSC, TMIN, TMAJ, PECM1, PECM2, PECM3, LD1, LD2, LD3, CORB, TRI Long, TRI Lat, TRI Med, ANC, SUP, BIC Long, BIC Short, BRA, BRD, ECRL, ECRB, ECU, FCR, FCU, PL, PT, PQ.	LP: 10 Hz.	NMF	R^2^ reconstruction levels (0.80, 0.85, 0.90)	For the 32-muscle set, one additional synergy was required compared with the 12-muscle set. The core synergy similarity exceeded 0.97, and the extra synergies primarily captured forearm and wrist movement patterns.
Chen [123]	Simulations	Running.	SO, GL, MG, TA, BF, VM, VL, RF, GLU, GM; 43 EMAs.	HP: 4th-order 50 Hz, rectification, LP: 4th-order 10 Hz, normalization.	SNMF ANLS	VAF_glo_ ≥ 80–95% and VAF_loc_ ≥ 75% to define plausible synergy range; CIRR based on within- and between-subject stability to identify optimal number;	Four optimal synergies were identified from 10-channel sEMG/EMA, whereas five optimal synergies were identified from 43 EMAs; the additional synergy involved deep/small muscles, particularly during early swing.
Camardella [124]	Human participants (N = 9)	Perform isometric contractions with the upper limb in position, grabbing the handle in 5 sites of the horizontal workspace.	FD, ED, BIC Long, BIC Short, BRA, TRI Lat, TRI Long, DELT1, DELT2, DELT3, PECM, INFRA, TRAPS, LD, TMAJ	BP:20–400 Hz, LP: 4 Hz.	NMF	Pearson corr, RMSE, R^2^	The global optimal set comprised 8 muscles, whereas subject-specific sets contained 6. Force estimation performance was comparable to that of the full muscle set (*p* > 0.05). Synergy similarity ranged from r = 0.74 to 0.78, and both pose-shared and pose-related synergy types were preserved.
Guez [125]	Human participants (N = 11)	Walking.	RF, VM, VL, BF, ST, TA, SO, GM, GL, ESL-Dom, ESL-Non, ESI-Dom, ESI-Non, MF-Dom, MF-Non.	BP: 20–400 Hz, LP: 10 Hz.	PCA, RFE, NMF	Synergy group coverage >80%; MLP validation accuracy	RFE-selected muscles covered more than 80% of the synergy groups. RFE outperformed PCA, achieving a 1.81% higher accuracy. Rectus femoris and trunk muscles (erector spinae and multifidus) were consistently selected, but the data-driven approach disregarded physiological constraints.
Kieliba [129]	Human participants (N = 8)	3D reaching.	DELT1, DELT2, DELT3, PECM, LAT, INFRA, RHO, PT, BIC Short, BIC Long, BRA, TRI Lat, TRI Long, TRAPS, TRAPM.	BP:50–500, 20–500 Hz, LP: 10, 0.5, 4, 20 Hz, Normalized.	FA	R^2^ ≥ 80%	Increasing the LP filter cutoff could decrease the number of synergies and could influence individual muscle contributions; extreme smoothing enhanced the comparison between active and inactive muscles, but it had an unclear impact on inter-subject similarity.
Zhao [130]	Human participants (N = 135)	Running.	GM, GLU, TFL, RF, VM, VL, ST, BF, TA, PL, GM, GL, SO	BP: 20–450 Hz, LP: 2–40 Hz.	NMF	VAF VAF/R^2^	EVAF proved to be the most robust metric, with VAF exceeding R^2^. It is recommended to combine EVAF with MAX, AVE, or UVA normalization and a low-pass filter cutoff around 10 Hz. Normalization had a greater effect on synergy structure than the filter cutoff frequency.
Ortega- Auriol [131]	Method studies	Gait analysis.	GLU, GM, BF, VL, RF, GL, TA, SO	BP: 10–500 Hz, LP: 20 Hz.	NMF	VAF > 90%	Normalization influenced the number, structure, and timing of synergies. TSM and ITM yielded the most consistent results, whereas UV was excluded because its VAF fell below 90%. Raw data and TSM produced four synergies, while WTM, ITM, and MP resulted in five synergies.
Ghislieri [132]	Human participants (N = 22)	Walking.	LD-R, LD-L, TFL, GM, RF, LH, MH, VM, GL, PL, SO, TA.	HP: 35 Hz, Demeaned, LP: 12 Hz.	NMF	VAF > 90%	Novel preprocessing method that makes the extraction of muscle synergies from walking tasks more robust and interpretable.
Oliveira [133]	Human participants (N = 12)	Treadmill walking.	TA, SO, GL, GM, VL, VM, RF, BF, ST, GLU	BP: 10–500 Hz.	NMF	VAF > 90%	Five motor modules accounted for most muscle activation in walking, and the identification of motor modules was similar to both types of data structures.
Colamarino [134]	Algorithm development study	The Box and Block Test performed 20 times with each hand.	ED, FDS, TRI Lat, BIC Long, DELT1, DELT3, PECM, TRAPS.	BP: 50–500 Hz, LP: 1, 10, 20 Hz,	NMF	VAF_glo_ > 0.9; VAF_loc_ > 0.7; VAF_diff_ < 0.05	Three synergies were sufficient to explain the Box and Block Test performance. The optimal configuration was a 10 Hz low-pass filter with no normalization. Synergy 1 (involving anterior and lateral deltoid) was associated with reaching, Synergy 2 (trapezius and biceps) with elevation and flexion, and Synergy 3 (anterior deltoid, pectoralis, and biceps) with the return phase. Inter-subject similarity exceeded 0.80.

Corr: Correlation Coefficient; RMSE: Root Mean Square Error; MLP: Multilayer Perceptron; EVAF: Error VAF; AVE: Average (normalization method); UVA: Unnormalized Variance Accounted (normalization method); TSM: Total Sum Method; MAX: Maximum Value Normalization; MED: Median Value Normalization; UAM: Unit Amplitude Normalization; ITM: Integrated Time Method; UV: Unit Vector; WTM: Wavelet Transform Method; MP: Maximum Peak; MCID: Minimal Clinically Important Difference; FWHM: Full Width at Half Maximum; CoA: Coefficient of Activation; ANLS: Alternating Nonnegative Least Squares; sMLE: sparse Maximum Likelihood Estimation; VAF_diff_: Increment in Global VAF after Adding one Synergy.

### 5.3. EMG Preprocessing

Across the reviewed methodological studies, EMG preprocessing consistently influenced synergy-related outcomes, although the magnitude and direction of these effects varied according to the preprocessing procedure and the specific outcome evaluated. Studies focusing on filtering generally indicate that cutoff frequency can alter reconstruction metrics, muscle contributions, and the estimated number of synergies, although the effects are not quantitatively consistent across datasets. As shown in Table 4, regarding the effect of different EMG preprocessing techniques, Shuman et al. [30] demonstrated that Total VAF was particularly sensitive to LP filtering, unit-variance normalization, and noise level, whereas synergy weights and activation profiles were less affected; consequently, these preprocessing choices could also influence the estimated number of synergies. Kieliba et al. [129] likewise showed that changing the LP cutoff altered individual muscle contributions and tended to affect the estimated number of synergies. However, the exact change in synergy number was not consistently reported across these studies. Taken together, these findings support sensitivity of synergy extraction to filtering, but they do not establish a uniform cutoff-dependent effect or a universally optimal filter setting.

Normalization appears to represent another important source of variability, and recent comparative evidence suggests that its influence may be as large as, or in some datasets greater than, that of moderate changes in filter cutoff. Banks et al. [24] found that trial-by-trial unit-magnitude normalization produced marked differences in synergy-vector variability, whereas neural-command variability was less sensitive to normalization and output normalization had relatively little influence. In a larger evaluation involving 135 participants and 225 combinations of preprocessing and factorization procedures, Zhao et al. [130] reported that normalization strategy had a greater influence on extracted synergy structure than LP cutoff frequency. Ortega-Auriol et al. [131] further showed that different amplitude-normalization strategies produced a difference of approximately one synergy under the tested conditions, and unit-variance normalization failed to satisfy the prespecified VAF criterion. These findings provide convergent evidence that normalization can materially influence synergy structure and model order, but the effect remains dependent on the normalization method and analytical outcome.

Evidence for other preprocessing and data-organization strategies is less uniform. Ghislieri et al. [132] reported that extracting principal activations as a preprocessing step improved the robustness and interpretability of synergies during walking. By contrast, Oliveira et al. [133] found relatively similar motor modules when comparing different EMG data structures, including single-trial, averaged, and concatenated data, suggesting a comparatively limited effect of data organization in their locomotor dataset. Colamarino et al. [134] further identified a 10 Hz LP filter combined with non-normalized EMG as a favorable configuration for the Box and Block Test, with three synergies sufficient to characterize the task. However, such task-specific optimization should not be interpreted as evidence for a universally optimal preprocessing pipeline, because the evaluated tasks, populations, and outcome criteria differed across studies.

Overall, the available evidence indicates that preprocessing is an important source of methodological variability in muscle synergy analysis, but its effects are outcome- and context-dependent rather than uniform. Filtering, normalization, and data organization may differentially affect reconstruction metrics, synergy number, synergy structure, and trial-to-trial variability. Among the reviewed studies, normalization shows relatively consistent evidence of influencing synergy-related outcomes, whereas the effects of filtering and data organization are more heterogeneous and task-dependent. Therefore, preprocessing parameters should be selected according to the task and analytical objective, applied consistently within a study, and reported in sufficient detail to support reproducibility and cross-study comparison.

### 5.4. Extraction Techniques Using Linear and Nonlinear Methods

Comparative studies of conventional factorization methods do not identify a single algorithm that consistently performs best across all datasets, tasks, and evaluation criteria. As shown in Table 5, considering how matrix-factorization techniques affect muscle synergies, Tresch et al. [105], Ebied et al. [59] and Rabbi et al. [60] compared several commonly used methods, including NMF, PCA, ICA, FA, and second-order blind identification (SOBI). Collectively, these studies showed that algorithm performance depends strongly on data characteristics, such as channel number, sparsity, noise level, component correlation, and task dynamics. NMF often performed favorably, particularly when its non-negativity assumption was compatible with the data and the number of synergies was smaller than the number of recorded channels. However, low-amplitude or highly correlated synergies remained difficult to identify, and some alternative methods performed poorly under limited-channel conditions. These findings therefore support NMF as a practical and widely applicable method, but not as a universally superior approach.

A second body of evidence indicates that the performance of NMF can be substantially affected by initialization, biomechanical constraints, and inter-individual variability, thereby motivating the development of modified factorization strategies. Soomro et al. [135] showed that sparse initialization improved reconstruction compared with random and SVD initialization under the tested conditions, whereas Ma et al. [35] reported improved repeatability and intra-subject consistency using MCR-ALS. Burkholder et al. [136] and Steele et al. [137] further demonstrated that biomechanical and task constraints, particularly correlated synergy activations, can reduce the accuracy with which factorization algorithms recover the underlying synergies. Other extensions have targeted different methodological limitations: Scano et al. [99] introduced mixed matrix factorization to jointly analyze muscle and kinematic variables, Delis et al. [138] developed an NMF-based approach to improve the categorization of spatial and temporal modules, and Shourijeh et al. [139] reported greater cross-subject consistency using concatenated NMF. Taken together, these studies suggest that algorithmic modifications can improve specific properties such as repeatability, multimodal integration, or cross-subject consistency, but the observed advantages are linked to the particular limitation each method was designed to address and should not be interpreted as evidence of universal superiority.

Nonlinear and autoencoder-based approaches extend synergy analysis beyond the assumptions of conventional linear decomposition, particularly when nonlinear interactions or inhibitory contributions are of interest. Studies using unsupervised or constrained autoencoders [41,98,140] showed that nonlinear latent representations can capture agonist–antagonist relationships or negative components that are not explicitly represented by standard nonnegative factorization. However, comparative evidence remains mixed. Zhao et al. [141] found that although autoencoders outperformed some linear approaches, NMF and ICA achieved higher reconstruction and classification accuracy in their tested datasets. Similarly, Buongiorno et al. [142,143] reported that autoencoder-based methods improved task-oriented joint-moment prediction or motor-intention decoding, but these gains were not always accompanied by superior EMG reconstruction. Thus, nonlinear models may provide advantages for particular functional objectives but improved predictive performance does not necessarily imply better reconstruction or greater physiological interpretability.

Application-oriented studies further illustrate that advanced methods may be advantageous under specific clinical, multimodal, or noise-related conditions. Lee et al. [144] applied autoencoder-based synergy analysis to distinguish spatial and temporal abnormalities during curved walking in individuals with stroke, whereas Russo et al. [145] used mixed matrix factorization to jointly represent EMG and unconstrained kinematic data. Rusta et al. [146] reported strong reconstruction performance and noise robustness for a deep-reinforcement-learning-based dynamic neuro-fuzzy system (DRL-DNF) in 24 manual wheelchair users. However, these results were obtained in specific populations and task settings and therefore require independent cross-dataset validation before broader conclusions can be drawn. Their contribution is better interpreted as demonstrating application-specific potential rather than establishing a new general-purpose standard for synergy extraction.

Recent studies also emphasize that reconstruction accuracy alone is insufficient for evaluating synergy algorithms. Berger et al. [147] incorporated force reconstruction into the factorization objective and showed that force-constrained NMF could outperform standard NMF in force-related tasks and under different noise conditions. Pan et al. [148] used network analysis to characterize relationships among primitive synergies across simple and complex upper-limb tasks, extending evaluation beyond vector similarity alone. Brambilla et al. [149], in a comparison of 16 autoencoder architectures, demonstrated a clear trade-off between reconstruction accuracy and physiological interpretability: architectures with the best reconstruction did not necessarily produce the most physiologically plausible synergies. Collectively, these findings show that algorithm evaluation should consider multiple dimensions, including reconstruction fidelity, task relevance, robustness, and physiological interpretability, rather than relying on a single performance metric.

Overall, the available evidence supports a context-dependent approach to algorithm selection. Conventional methods such as NMF remain widely used and interpretable, but their performance is sensitive to initialization, noise, channel configuration, signal correlation, and task structure. Modified, mixed, nonlinear, and autoencoder-based methods may improve specific outcomes, including repeatability, multimodal integration, task prediction, or robustness, but these advantages are not consistently observed across datasets or validation criteria.

**Table 5:** Experimental studies investigating muscle synergies in synergy methodology.

Study	Study Category (Sample Size N = Subject Number)	Methods				Outcomes
		Recorded Muscles	EMG Preprocessing	Synergy Extraction	Analysis Criteria	
Ma [35]	Algorithm development study	DELT1, DELT3, ECR, FCR, BRD, BB, TRI.	BP: 40–400 Hz, Rectified, LP: 5 Hz.	NMF, SMMA, MCR-ALS.	Not reported	MCR-ALS showed a significant advantage in repeatability and intra-subject consistency and was more beneficial to the reliability of the motor function evaluation.
Ebied [59]	Algorithm development studies	Not reported	Not reported	PCA, NMF, ICA, SOBI.	Not reported	The SOBI algorithm was a proposed alternative to NMF when the number of channels was low; NMF was more suitable to extract muscle synergies when the number of channels was higher than the number of synergies.
Rabbi [60]	Algorithm development study	GM, GL, SO, PL, Sar, TA, VL, RF, BF, PB.	BP: 30–400 Hz, Rectified, LP: 6 Hz, Normalized.	PCA, FA, ICA, NMF.	VAF > 85%	NMF was a more suitable factorization technology for identifying muscle synergies from muscle contraction under dynamic tasks performed at different movement speeds.
Tresch [105]	Algorithm development study	ST, SA, RI, AM, VI, SM, VE, BF, IP.	Not reported	PCA, FA, ICA, NMF.	Not reported	Most algorithms performed well in identifying the synergy subspace and the synergy vectors (except PCA). Synergies activated with low amplitude or correlated with each other were difficult to identify.
Soomro [135]	Algorithm development study	GM, BF, MG, SO, RF, VM, VL, TA.	BP: 20–400 Hz, Rectified, LP: 5 Hz.	NMF	Not reported	Sparse initialization performed significantly better than all other kinds of initialization in reconstructing muscle synergies, regardless of the correlation level in the data.
Burkholder [136]	Simulations	A three-degree-of-freedom version of a 31-muscle cat hindlimb model.	Not reported	NMF	VAF	Indicated that mechanical constraints led to the muscle synergies being unrecognizable.
Steele [137]	Algorithm development study	DELT1, DELT2, DELT3, SUPSP, INFRA, SUBSC, TMIN, TMAJ, PT, PECM1, PECM2, PECM3, LAT1, LAT2, LAT3, CORB, TRI Long, TRI Lat, TRI Med, ANC, SUP, BIC Long, BIC Short, BRA, BRD, ECRL, ECRB, ECU, FCR, FCU.	Not reported	PCA, fICA, PCA ICA, NMF, pICA.	Not reported	Correlation of synergy activations decreased the ability of these matrix algorithms to accurately identify muscle synergies; Although the accuracy of muscle synergies is influenced by biomechanical and task constraints, the robust experimental protocol could be applied to capture more comprehensive variability of muscle activations for improving it.
Delis [138]	Algorithm development study	ED, BRD, TRI Med, TRI Lat, DELT1, DELT3, PEC, BB.	HP: 20 Hz, Rectified, LP: 450 Hz, Normalized.	sNM3F	Not reported	The method can effectively achieve better task module categorization.
Shourijeh [139]	Algorithm development study	RF, VM, VL, ST, BF, MG, GL, TFL.	BP: 20–450 Hz, Rectified, LP: 6 Hz, Normalized.	NMF	VAF	CNMF was a more dependable method than NMF and improved the consistency between subjects. Thus, compared to the muscle synergies between subjects, CNMF should be considered a priority.
Zhao [141]	Algorithm development study	Simulated data: 6, 8, 10, 12 channels; NinaPro data: 10 upper limb muscles.	Simulated data: Added Gaussian noise (SNR 10, 15, 20 dB).	PCA, ICA, FA, NMF, Autoencoder.	VAF	NMF and Independent Component Analysis performed best. Autoencoder outperformed Factor Analysis in VAF and Principal Component Analysis in similarity but was inferior to NMF and Independent Component Analysis. SNR and Channel Number significantly affected results. NMF and Principal Component Analysis achieved higher classification accuracy; K-Nearest Neighbors and Random Forest outperformed Linear Discriminant Analysis and Support Vector Machine.
Buongiorno [142]	Human participants (N = 9)	BIC Short, BIC Long, BRD, TRI Long, TRI Lat, DELT1, DELT3, TRAPS, PECM, TMAJ, INFRA, LAT, RHO.	BP: 5–500 Hz, 50 Hz notch; HP 20 Hz (2nd order Butterworth), rectification, LP 5 Hz (2nd order Butterworth), normalized to max per channel	Autoencoder, NMF	Multivariate R^2^, RMS Error	Autoencoder’s EMG reconstruction was slightly lower than NMF, but Autoencoder significantly outperformed other synergy-based models in joint moment prediction (task space), achieving performance comparable to the direct EMG-to-moment mapping model.
Buongiorno [143]	Human participants (N = 6)	BIC Short, BIC Long, BRD, TRI Long, TRI Lat, DELT1, DELT3, TRAPS, PECM, TMAJ, INFRA, LAT, RHO.	HP: 20 Hz (2nd order Butterworth), rectification, LP 5 Hz (2nd order Butterworth), normalized to max value	Autoencoder, NMF.	Multivariate R^2^, RMS Error	Autoencoder significantly outperformed NMF in muscle activation reconstruction (R^2^, *p* = 0.028). For joint moment estimation, Autoencoder showed no significant difference from the Electromyography-to-moment mapping model and outperformed the synergy-based model. It demonstrated the potential of Autoencoder for motor intention detection.
Lee [144]	Human participants (N = 13)	RF, AM, VM, TA, GM, ST, BF, MG.	BP: 20–750 Hz; HP: 35 Hz (4th order Butterworth), rectification, LP: 5 Hz (4th order Butterworth). Segmented to 200 points, normalized to peak.	Autoencoder	VAF, Pearson correlation.	During curved walking in stroke patients, spatially, Synergy 3 (early swing) had the lowest similarity during Outside Curved Walking, and Synergy 4 (late swing) had the lowest during Inside Curved Walking. Temporally, peak timings of Synergy 1 and 2 were significantly delayed during Outside Curved Walking, and Synergy 1 and 3 were delayed during Inside Curved Walking. Specific spatiotemporal pathological synergy features were revealed depending on curve direction.
Russo [145]	Algorithm development study	14 upper limb muscles (shoulder and elbow muscles during reaching tasks)	Tonic removal (user-adjustable: EMG LP: 20 Hz, kinematic LP: 10 Hz)	MMF, NMF, PCA	R^2^	MMF jointly factorizes non-negative EMG signals and unconstrained kinematic variables, thus directly linking muscle synergies to task-space coordinates. The Synergy Analyzer toolbox, which implements this method, has been made available as an open-source resource.
Rusta [146]	Algorithm development study	DELT1, DELT3, PECM, BIC Long, TRI Lat, TRI Long.	BP: 10–500 Hz, Rectified, LP: 6 Hz,	DRL-DNF, NMF, ICA, Autoencoder	VAF > 90%	DRL-DNF achieved a variance accounted for (VAF) of 94.12 ± 4.12% under noise-free conditions and 90.1 ± 4.18% under noisy conditions with three synergies, outperforming NMF, ICA, and autoencoders, while also better capturing nonlinear agonist–antagonist interactions.
Berger [147]	Algorithm development study	Simulated data + EMG data from isometric reaching tasks.	Not reported	FCNMF, NMF	Force reconstruction accuracy	FCNMF incorporated a force reconstruction error penalty into its cost function, enabling it to outperform standard NMF across various noise levels and to accurately reconstruct force trajectories even in participants where standard NMF had failed.
Pan [148]	Human participants (N = 28)	TRAPS, PECM, DELT1, DELT2, DELT3, BIC, BRD, TRI Lat, TRI Long.	HP: 50 Hz, LP: 10 Hz.	NMF	VAF > 95%	Four primitive synergies explained over 95% of EMG variance in basic tasks. Complex tasks were achieved through different combinations of these primitives. Muscle synergy network topologies differed significantly across tasks.
Brambilla [149]	Algorithm-development study	ERS, TMAJ, INFRA, LT, MT, UT, DELT1, DELT2, DELT3, PT, TRI Long, TRI Long, TRI Lat, BIC Long, BIC Short, BRA.	BP:20–450 Hz, LP:6 Hz.	Autoencoder, NMF	RMSE, R^2^	The ReLU-tanh activation combination produced synergies most similar to those from NMF (similarity = 0.96, correlation = 0.96). In contrast, the linear encoder yielded the best reconstruction accuracy but resulted in uninterpretable synergies. Autoencoders were found to be highly sensitive to initialization, and the best reconstruction performance did not necessarily correspond to physiological interpretability.

ReLU: Rectified Linear Unit; tanh: Hyperbolic Tangent; RMSE: Root Mean Square Error; fICA, Fast Independent Component Analysis; sNM3F: Sample-Based Non-Negative Matrix Tri-Factorization; pICA, Probabilistic ICA. FCNMF: Force-Constrained Nonnegative Matrix Factorization. SMMA: Self-modeling Mixture Analysis. CNMF: Concatenated Non-negative Matrix Factorization.

### 5.5. Choosing the Synergy Number

Although VAF remains the most widely used reconstruction-based criterion for determining the number of synergies, the reviewed evidence does not support a single universal threshold. The absence of a consistent and objective criterion remains a major methodological challenge in muscle synergy analysis [33] and VAF thresholds vary substantially across studies [150]. A threshold such as VAF > 90% has frequently been adopted to ensure adequate reconstruction and has also been used to characterize population-level distributions of synergy number [89]. However, high explained variance does not necessarily indicate that all task-relevant information has been captured. Barradas et al. [150] showed that residual EMG components remaining after 90% of the variance had been explained could still contain movement-related information, while other studies have similarly suggested that residual components may reflect additional synergy-related structure [151]. Thus, VAF should be interpreted as a measure of reconstruction performance rather than as direct evidence that the selected model order fully represents the underlying motor-control organization.

The reliability of VAF-based model-order selection is further constrained by its sensitivity to inter-individual variability, preprocessing, noise, and task-space sampling. Kim et al. [17] showed that applying a single VAF threshold across individuals could lead to inconsistent estimates of synergy number, motivating the use of additional statistical procedures such as intra-class measures, between-level correlation analysis, and clustering. Task sampling is another important source of variation: Augenstein et al. [28] found that reducing the number of reaching directions altered VAF-based results and the validity of the extracted synergies. In addition, functional organization in task space should be considered when evaluating the adequacy of a synergy solution [138]. Collectively, these studies indicate that a fixed VAF threshold alone cannot distinguish methodological variation from biologically meaningful differences and should therefore be complemented by additional criteria or sensitivity analyses.

Alternative model-order criteria have been proposed to reduce reliance on arbitrary VAF cutoffs, but their advantages are also context-dependent. Ballarini et al. [152] developed the method of choosing the optimal number of muscle synergies (ChoOSyn) and reported performance comparable to VAF-based approaches in simulated data, together with improved classification performance and error-related measures in the evaluated real datasets. Ranaldi et al. [110] proposed an AIC-based approach that showed good model-order identification under the tested conditions, whereas MDL has been reported to perform better when the channel-to-synergy ratio is relatively high [59]. These approaches provide more explicit consideration of model complexity than fixed reconstruction thresholds, but their relative performance depends on data structure, channel configuration, noise assumptions, and the statistical formulation used. Therefore, current evidence supports their use as complementary model-selection tools rather than as universally superior replacements for VAF.

Task demands themselves may also change the number of synergies required to represent motor behavior, as shown in Table 6. Herzog et al. [153], using an R^2^ knee-point criterion with bootstrap analysis in 36 healthy men, found that more synergies were required following adaptation to a force field than at baseline, indicating that model order can change with altered control demands. Kuska et al. [154] similarly showed in a two-dimensional musculoskeletal simulation that models containing more synergies tolerated greater simulated knee-extensor weakness. However, this simulation provides indirect evidence concerning the functional consequences of model complexity and does not establish that a larger synergy number is universally associated with greater physiological robustness. Together, these findings suggest that the number of synergies may reflect both task demands and the model-selection procedure used to determine it.

Data organization and population characteristics introduce further heterogeneity. Zhao et al. [155] showed in 15 healthy participants that concatenated, averaged, and other trial-processing approaches produced different estimates of synergy number during multidirectional reaching; the concatenated approach yielded the greatest number of synergies and the averaged approach the fewest, leading the authors to recommend at least five trials and four reaching directions under their experimental conditions. Population-related effects may be less evident in total synergy number than in synergy organization. Saito et al. [156] found only a small, non-significant difference in median total synergy number between preschool and school-aged children, despite observing two additional synergy clusters in the older group. Similarly, Kian-Bostanabad et al. [157] identified six synergies during six-direction maximum voluntary isometric contraction (MVIC) but only two during maximum voluntary isometric extension (MVIE) in 14 healthy men using a five-criterion model-order rule. These findings indicate that task type, data organization, and population characteristics can influence different aspects of synergy organization, and that the total number of synergies alone may not capture all biologically or methodologically relevant differences.

Overall, VAF remains useful for reconstruction-based assessment, but no universal threshold is supported across populations, tasks, and preprocessing conditions. Alternative methods such as ChoOSyn, AIC, and MDL may reduce reliance on fixed VAF cutoffs, yet their performance also depends on dataset and model assumptions. Moreover, task demands, trial organization, and population characteristics can alter either the estimated number of synergies or the organization of the extracted modules. Accordingly, the estimated number of synergies should be reported together with the model-selection criterion, relevant preprocessing and data-organization procedures, and, where feasible, complementary stability and sensitivity analyses to enhance the interpretability and comparability of findings across studies.

### 5.6. Feasible Methodological Recommendations

Muscle synergy analysis has been increasingly applied in motor control, clinical, rehabilitation, and engineering research. However, heterogeneity in EMG acquisition, preprocessing, factorization, model-order determination, and validation continues to limit cross-study comparability and reproducibility. Based on the methodological and experimental evidence summarized in Table 3, Table 4, Table 5 and Table 6, we propose several evidence-informed recommendations covering three major stages of the muscle synergy analysis pipeline: (1) data acquisition and preprocessing, (2) synergy extraction and model selection, and (3) validation and application-oriented approaches.

As summarized in Table 7, the strength of evidence varies across methodological factors. Available evidence indicates that muscle selection, filtering and normalization, trial organization, and task design can influence extracted muscle synergies. In contrast, universal recommendations for model-order thresholds, information-theoretic criteria, reduced muscle sets, and advanced factorization methods remain limited or strongly context-dependent. Therefore, these recommendations emphasize transparent reporting, methodological consistency, complementary model-evaluation criteria, and task-specific validation.

(1) Data acquisition and preprocessing: Reliable muscle synergy analysis depends on the quality and consistency of EMG acquisition and preprocessing. Muscle selection should reflect task biomechanics, anatomical coverage, and the study objective. Reduced or data-driven muscle sets may reduce recording dimensionality, but their suitability should be validated for the specific task and population because reducing the number of recorded muscles can affect VAF, synergy number, and task-specific muscle contributions. Filtering, normalization, trial organization, electrode placement, and other acquisition parameters should also be clearly reported and consistently applied. Rather than imposing a universal protocol, minimum reporting standards are recommended to improve transparency and cross-study comparison. The availability of open raw and processed EMG datasets, together with clearly defined evaluation metrics and processing procedures, may further enhance reproducibility and facilitate methodological comparisons.

(2) Synergy extraction and model selection: Factorization algorithms, initialization strategies, and model-order criteria should be selected according to the research objective and dataset characteristics. No single factorization method has been shown to be optimal across all conditions, because performance can vary with signal structure, channel number, noise level, component correlation, and task characteristics. Initialization procedures should therefore be reported and repeated, or alternative initializations may be considered when solution stability is important. Advanced factorization frameworks, including probabilistic/Bayesian, tensor-based, nonlinear, and deep latent-variable approaches, may offer advantages for representing uncertainty, multidimensional structure, or nonlinear neuromuscular relationships. However, these advantages remain context-dependent and should be evaluated in terms of interpretability, robustness, and computational cost. Model order should likewise not be determined solely using a fixed VAF or R^2^ threshold. Complementary criteria and sensitivity analyses should be considered where appropriate, and the statistical assumptions underlying information-theoretic criteria should be clearly reported.

(3) Validation and application-oriented approaches: Extracted muscle synergies should not be evaluated solely on the basis of reconstruction performance. Reliability and repeatability should be assessed when synergy stability is an important outcome, with particular attention to electrode placement and between-session acquisition consistency. Because synergy organization can also vary with task type and experimental constraints, the extracted modules should be interpreted within the specific task context in which they were obtained. Their functional relevance may be further examined by comparing synergy-related outputs with force, kinetic/kinematic measures, musculoskeletal-model predictions, or other task-relevant physiological observations. For muscles that can be experimentally measured, agreement between simulation-derived activation patterns and recorded EMG signals can provide additional support for the physiological plausibility of model-based estimates. Musculoskeletal simulation can also extend synergy analysis to small and deep muscles that are difficult to assess using conventional surface EMG, thereby providing a more comprehensive representation of neuromuscular coordination. However, such agreement provides only indirect and complementary evidence for physiological plausibility and does not independently demonstrate that the extracted modules represent genuine neural control primitives. In particular, for muscles that cannot be directly measured, it remains difficult to experimentally verify the accuracy of their estimated activations and their contributions to specific synergy modules.

## 6. Conclusions

Muscle synergy theory has been widely applied in neuroscience, robotics, sports science, and rehabilitation to investigate motor control and coordination strategies and to characterize functional impairments associated with pathological conditions. However, substantial methodological variability across studies remains a major challenge for interpreting and comparing reported synergy outcomes. This review highlights key methodological factors affecting the robustness and reliability of muscle synergy analysis, including experimental design, EMG preprocessing, synergy extraction approaches, model-order selection, and validation strategies.

The findings of this review suggest that improving the robustness and reliability of muscle synergy analysis may improve interpretation and broader application. Methodological criteria and emerging approaches should therefore be further refined and validated across different tasks, populations, and analytical settings. For example, experimental protocols may include multiple degrees of freedom and sufficiently diverse tasks to capture representative muscle activation patterns and characterize the relevant synergy subspace. In addition, open-source musculoskeletal modeling platforms such as OpenSim [158] may provide a useful complementary tool for sensitivity analysis, evaluation of muscle-selection strategies, and assessment of biomechanical plausibility. However, musculoskeletal simulation provides only indirect and complementary support for the physiological plausibility of the extracted modules and cannot independently demonstrate that these modules correspond to genuine neural control primitives. Although simulation can extend synergy analysis to experimentally inaccessible muscles, its results should be interpreted together with experimental EMG and other physiological evidence when inferring the underlying neuromuscular control mechanisms. Overall, improved methodological rigor and transparent reporting may enhance the reproducibility, interpretability, and broader application of muscle synergy analysis across neuroscience, rehabilitation, and robotics.

## Figures and Tables

**Table 1:** Definitions and primary quantitative criteria for core concepts in muscle synergy analysis.

Term	Formal Definition	Used Quantitative Criteria	Suggested Operational Criteria	Notes
Robustness	The ability of the extraction algorithm to maintain stable synergy structures despite external perturbations, signal noise, or biomechanical constraints, e.g., [16,22].	Relative change in reconstruction error; coefficient of variation (CV) across perturbations; bootstrap confidence intervals; similarity of synergy vectors across repeated perturbations.	Robustness may be considered acceptable when perturbations produce only small changes in the extracted solution. These thresholds should be predefined according to the analysis context.	No universally accepted threshold exists across all synergy methods; perturbation type and metric should be reported explicitly.
Reliability	The overall consistency and dependability of the EMG acquisition and factorization pipeline to yield stable outcomes, e.g., [35].	ICC; CV; standard error of measurement (SEM).	ICC < 0.50: poor; 0.50–0.75: moderate; 0.75–0.90: good; >0.90: excellent reliability. CV < 10% may indicate low relative variability, although acceptable values depend on the outcome and task.	ICC model, confidence interval, and CV calculation should be reported.
Repeatability	The degree of agreement between synergy outputs obtained under identical conditions, e.g., [36,37,38,39,40].	Pearson correlation coefficient; Cosine similarity.	High repeatability may be indicated by strong correlations or similarity values, for example, r ≥0.80 or cosine similarity ≥0.90, together with small between-trial variability.	The selected threshold should be justified because similarity metrics are influenced by normalization and matching procedures.
Reproducibility	The degree of agreement between synergy outputs obtained under changed conditions (e.g., inter-session or inter-subject), e.g., [41].	Cross-day or cross-subject scalar products; Z-score normalized variance comparison.	Reproducibility may be supported by good-to-excellent ICC, high synergy similarity, etc.	Cross-subject reproducibility should be interpreted more cautiously because biological variability may be substantial.
Physiological Validity	The extent to which extracted mathematical modules represent genuine neural control strategies rather than algorithmic artifacts.	Association with biomechanical variables; prediction of unmeasured muscle activations.	Similarity between predicted muscle activations and measured sEMG envelopes from a limited set of muscles.	Successful simulation or prediction provides indirect support for physiological plausibility but does not independently demonstrate neural control primitives.

Note: The numerical values provided in the table are intended as practical reference criteria rather than universal thresholds.

**Table 2:** Methodological factors affecting synergy extraction.

Steps	Factor	Cause	Main Effect	Potential Solutions
Experimental constraints	Task design	Limited movements, sex, age, motor habits, experimental settings.	Variations in EMG signals are induced by task, individual, or environmental factors.	Multi-task and multi-condition acquisition. Inter-subject and environmental variability. Averaging or combining multiple trials. Multiple task subspaces.
EMG Acquisition	Subjects/Electrode placement/Signal noise	Skin condition, electrode placement, test conditions, cross-talk, inter-study variability, spatial variability, time, resources, motion artifacts, EMG equipment limits, electrophysiological signals and external noise.	Variable SNR amplitude, signal variability.	Follow SENIAM guidelines and standardize setup. Ensure consistent electrode placement and record from muscle belly. Extract synergies based on activation timing.
Muscle Selection	Muscle subset/Difficulty accessing deep/small muscles	Biomechanical constraints, incomplete synergy sets, loss of precision control.	Incomplete synergy representation.	Include as many functionally relevant muscles as feasible. Dominant/Major muscles. Simulations or model-based to obtain more muscle activities.
EMG signal processing	Raw-EMG filtering	Motion artifacts, low-frequency drift, and high-frequency noise. Raw EMG is commonly band-pass filtered.	Alters the frequency content and signal quality of raw EMG; inappropriate filtering may distort subsequent synergy estimates.	Report filter type, order, and cutoffs. Apply baseline correction when needed; use channel rejection or segment masking for severe artifacts.
	Full-wave rectification	Bipolar raw EMG contains positive and negative signal values.	Converts the filtered EMG into a positive signal representing muscle activity magnitude.	Apply full-wave rectification after raw-EMG filtering and before envelope extraction.
	Envelope generation	LP cutoff: approximately 2–15 Hz.	Determines the temporal smoothness and resolution of activation profiles; may affect synergy weights and activation coefficients.	Select the cutoff according to task dynamics and analytical objectives; distinguish envelope filtering from raw-signal filtering.
	Temporal normalization	Trials, sessions, and subjects may differ in movement duration and event timing.	Aligns activation profiles across movement phases and enables comparison between trials or participants.	Align signals to defined kinematic events and normalize time to a common scale, such as 0–100% of the movement cycle.
	Amplitude normalization	EMG amplitude varies across muscles, subjects, and sessions. Options include SELF, MVC, median trial maximum, and unit-variance scaling.	Changes the relative contribution of muscles and may affect synergy weights, activation profiles, and the estimated number of synergies.	Select a method consistent with the study objective; report the normalization reference and apply it consistently across all signals.
Extraction algorithms	EMG decomposition	NMF, PCA, ICA, FA, MCR-ALS, MMF.	Each has assumptions and limitations; sensitive to initialization.	Autoencoder neural networks. Rectified latent variable model. Non-linearity model.
Synergy Number Determination	Reconstruction accuracy	VAF-based: fixed threshold, elbow, knee, linear fit; other: AIC, MDL, BIC.	No unified VAF or R^2^ cutoffs standard, leading to inconsistencies.	Avoid treating VAF cutoffs as universal defaults; tailor thresholds to task complexity and noise levels. Utilize objective, information-based criteria (e.g., AIC, MDL) or dual-criteria approaches, accompanied by sensitivity analyses. Cross-task and similarity analysis or robust metrics.

**Table 3:** Experimental studies investigated muscle synergies in different experimental constraints.

Study	Study Category (Sample Size N = Subject Number)	Tasks	Methods				Outcomes
			Recorded Muscles	EMG Preprocessing	Synergy Extraction	Analysis Criteria	
Torres- Oviedo [16]	Animal experiments (N = 6)	Postural perturbations.	GM, GLUP, GLUA, SA, ST, BFMA, BFMM, VL, VM, SO, BFMP, RF, PLAN, EDL, SEMA, ILPS, SEMP, TFL, GRAA, FDL, GRAP, TA, MG, SRTA, GL.	HP: 35 Hz, Demeaned, Rectified, LP: 30 Hz.	NMF	VAF_glo_ > 80%	A group of five functional muscle synergies was robust across a range of dynamic postural tasks as well as quiet stance and generalized across subjects.
Kim [17]	Human participants (N = 9)	Walking on the treadmill.	TA, MG, RF, MH.	HP: 35 Hz, Demeaned, LP: 12 Hz.	NMF	VAF	The method reliably identifies four or five synergies underpinning walking in unimpaired adults and reveals synergies’ poor reproducibility.
Munoz Martel [19]	Human participants (N = 15)	54 cycles of a specific task on stable ground and an unstable oscillating platform.	GM, MA, TFL, RF, VM, VL, ST, BF, TA, PL, GM, GL, SO.	HP: 50 Hz, Rectified, LP: 20 Hz.	NMF	R^2^	Addition of a new synergy with complementary function highlighted an increased motor output’s robustness in the presence of perturbations; Modularity might be an important feature to ensure robustness by supporting adaptive control strategies depending on the task characteristics.
Roh [22]	Human participants (N = 8)	Isometric 3D force target matching in three protocols using the MACARM robotic system	BRD, BB, TRI Long, TRI Lat, DELT1, DELT2, DELT3, PECM.	BP: 20–450 Hz.	NMF	VAF_glo_ > 90%	The composition of muscle synergies remained relatively consistent under biomechanical task conditions, experimental protocols, and subjects.
Scano [27]	Human participants (N = 14)	Upper limb reaching.	INFRA, LT, MT, UT, DELT1, DELT2, DELT3, PEC, TRI Lat, TRI Long, BIC Long, BIC Short, PT, BRD.	HP: 50 Hz, Rectified, LP: 10 Hz.	NMF	R^2^ > 80%	A reduced set of muscle modules remained identifiable despite substantial movement variability, and the repeatability and similarity of synergies were shared across subjects and sessions.
Augenstein [28]	Simulations	Planar reaching movements toward 36 directions.	DELT1, DELT2, DELT3, SUPSP, INFRA, SUBSC, TMIN, TMAJ, PT, CORB, PECM1, PECM2, PECM3, LAT1, LAT2, LAT3, TRI Long, TRI Lat, ANC, SUP, BIC Long, BIC Short, BRA, BRD.	Normalized to peak activation across all reaches.	NMF PCA	VAF_glo_ > 90%	The number and choice of reaching directions also affected the validity of the extracted muscle synergies. When the number of reaches was fewer than six, the muscle synergy composition would change; six reaching directions were required to obtain stable and representative synergy estimates.
Taborri [37]	Human participants (N = 8)	Walking, stepping in place, running, and stair ascent and descent.	TFL, RF, VL, VM, PL, TA, SO, GLU, ST, BF, LH, MH.	HP: 50 Hz, Rectified, LP: 20 Hz.	NMF	VAF_glo_ ≥ 90% VAF_loc_ ≥ 75%	The number of muscle synergies was not repeatable either within or between subjects and the variability of intrinsic sEMG signal and the electrode repositioning would affect the repeatability of muscle synergies rather than factorization algorithms; Synergy vectors and temporal activity patterns could be selected as powerful neurophysiological indices.
Shuman [40]	Clinical populations (N = 11)	Walking.	GM, LH, MH, VL, RF, SO, TA.	HP: 40 Hz, Rectified, LP: 4 Hz. Normalized.	NMF	VAF	Complexity and structure of muscle synergies were repeatable across days when EMG signals were averaged over multiple gait cycles.
Gentner [115]	Human participants (N = 8)	Reaching.	BRD, BIC Long, BIC Short, TRI Long, TRI Lat, DELT1, DELT2, DELT3, PECM1, TRAPM, LD, TMAJ, INFRA.	BP: 20–450 Hz, Rectified, LP: 5 Hz, Resampled to 100 Hz, Normalized.	NMF	R^2^ > 80%	Muscle synergy structure was robust throughout the visuomotor control adaptation process, suggesting that changes in muscle activation patterns arose from orientational adjustments of rotational muscle recruitment.
Geng [116]	Human participants (N = 24)	Four forearm movements under specific constraints as detailed in different experimental sessions.	ECU, BRD, FCU, ECR, FDS, EDC, PT, BIC (Long/Short), TRI, FPL, FCU, EPL.	BP: 30–450 Hz, Rectified, LP: 20 Hz.	NMF	VAF	The muscular compositions of forearm muscle synergies were slightly adjusted under variant force and arm position constraints.
Pale [117]	Human participants (N = 10)	Publicly available hand grasp NinaPro Database.	Forearm-specific muscles.	BP: 20–400 Hz, Rectified, Smoothed with LP filtering with a 100 ms wide moving average window.	NMF	VAF_glo_ (80, 85, 90, and 95%)	The repositioning of the virtual electrode significantly reduced the similarity of the synergy within the session and increased the similarity of the synergy extracted in different sessions; spatial similarity was significantly lower in inter-session than intra-session conditions.

VAF_glo_: the global VAF; VAF_loc_: the local VAF. All muscle abbreviations are listed in the Abbreviations table.

**Table 6:** Experimental studies investigating muscle synergies in synergy number.

Study	Study Category (Sample Size N = Subject Number)	Tasks	Methods				Outcomes
			Recorded Muscles	EMG Preprocessing	Synergy Extraction	Analysis Criteria	
Ranaldi [110]	Algorithm development study	Simulated data.	RF, VM, VL, TA, GLU, BF, SO, GM; BRD, BIC Short, BIC Long, TRI Lat, TRI Long, DELT1, DELT2, DELT3, PECM, TRAP, INFRA, TMAJ, LD.	Rectification, LP: (5, 10, 20 Hz).	NMF (Gaussian)	Modified AIC, VAF, R^2^	The modified AIC consistently and accurately determined the number of synergies across varying signal-to-noise ratios, filter settings, and datasets and outperformed conventional threshold-based rules.
Barradas [150]	Human participants (N = 15)	Reaching.	PT, BRD, BIC Long, TRI Lat, TRI Long, DELT1, DELT2, DELT3, PECM, MT.	BP: 20–450 Hz, HP and LP.	NMF	R^2^ > 90% synergy count, force reconstruction	Even when more than 90% of the variance was explained, the residuals still contained systematic information that influenced performance. Moreover, “easy” and “hard” virtual surgeries, despite sharing identical synergy-force mappings, yielded different initial errors attributable to residual effects.
Kim [151]	Human participants (N = 14)	Unexpected external perturbation.	SCM, EO, ESC, EST, ESL, ADD, RF, VM, TA, TFL, GLU, GM, BF, PL, MG, SO.	HP: 35 Hz, demeaned, rectified, LP: 40 Hz, normalized to maximum value.	NMF	VAF > 90%, k-means clustering	In athletes, distinct synergies were observed that activated ankle plantarflexors or neck extensors during the early perturbation phase, whereas non-athletes displayed co-activation of ankle agonists and antagonists.
Herzog [153]	Healthy participants (N = 36)	Reaching.	TRAPS, DELT1, DELT2, DELT3, LD, PECM, SAN, TRI Lat, TRI Med, BIC, BRD.	BP: 20–450 Hz, electrocardiogram removal, LP: 10 Hz.	NMF	R^2^, Bootstrap method	Baseline synergies (R^2^ = 0.82, 3.6 synergies) could not explain muscle patterns after adaptation. Adaptation required new synergy structures (1.92 synergies) and a four-phasic activation pattern. Adaptation-specific synergies facilitated retention and generalization.
Kuska [154]	Simulations	Tracking of normal gait with simulated muscle weakness and contracture to examine gait sensitivity.	HAM, GLU, IP, RF, VAS, GAS, SO, TA.	Not applicable.	NMF (fixed 3, 4, 5 synergies)	Sensitivity analysis across synergy counts (3, 4, 5)	Five-synergy control tolerated 40% more knee extensor weakness than four-synergy control and 51% more than three-synergy control. Reducing synergy number increased gait sensitivity to weakness and contracture.
Zhao [155]	Human participants (N = 15)	Point-to-point reaching movements.	ERS, TMAJ, INFRA, TRAPS, DELT1, DELT2, DELT3, PEC, TRI Long, TRI Lat, BIC Short, BRD, PT.	HP: 30 Hz, LP: 10 Hz.	NMF	VAF > 80%, ΔVAF < 5%	The concatenation approach yielded the highest number of synergies (3.87 ± 0.64), whereas averaging produced the lowest (2.40 ± 0.51). Accordingly, the authors recommend using at least five trials, four reaching directions, and the concatenation method.
Saito [156]	Human participants (N = 21)	Threw balls toward a fixed target.	INFRA, SUPSP, TRAPS, DELT1, DELT2, DELT3, SAN, PECM, BIC, TRI Long, BRD, FCU, ECRL, LD.	HP: 40 Hz, LP: 15 Hz.	NMF	VAF > 0.9; Gap Statistic; Hierarchical clustering	There was no significant difference in total synergy number between school-aged children (median = 6.5) and preschoolers (median = 6.0). However, the school-aged group exhibited more synergy clusters (8 vs. 6) and higher synergy sparseness.
Kian-Bostanabad [157]	Human participants (N = 14)	Maximum voluntary isometric contraction in six main directions with two repetitions and maximum voluntary isometric extension.	EO, RA, IO, ES, IL3, IL6, LD, MF.	BP: 10–500 Hz, LP: 10 Hz.	NMF	VAF > 90%, R^2^ > 80%, ΔVAF < 5%, ΔR^2^ < 5%, MSE < 5 × 10^−5^	Under MVIC conditions, six synergies were extracted, corresponding to one per principal direction, with an overall VAF of 99.65%. In contrast, only two synergies sufficed under MVIE conditions, achieving a VAF of 94.14%. These findings indicate that the number of synergies is primarily dictated by task type.

**Table 7:** Evidence underlying feasible methodological recommendations for muscle synergy analysis.

Methodological Stage	Representative Studies	Study Design	Task	Methodological Factor	Outcome Metric	Effect Direction/Main Evidence	Proposed Strength of Evidence	Evidence-Informed Recommendation
Data acquisition and preprocessing	[32,122,124,125].	Simulation and human experimental studies	Reaching, isometric upper-limb contractions, walking.	Number and selection of recorded muscles.	VAF/R^2^, synergy similarity, RMSE, force-estimation or classification accuracy.	Fewer recorded muscles may inflate VAF, whereas appropriately selected reduced muscle sets can preserve major synergy structures and functional performance; data-driven selection may nevertheless overlook physiological constraints.	Moderate	Select muscles according to task demands, anatomical coverage, and study aims. Validate reduced muscle sets before wider application.
	[24,30,129,130,131,134].	Human, clinical, methodological, and algorithm-development studies	Walking, gait analysis, 3D reaching, running, Box and Block Test.	Filtering and normalization.	VAF, R^2^, synergy number, synergy vectors/activations, inter-subject similarity.	Filter settings and normalization procedures altered VAF, synergy number, muscle contributions, and/or synergy structure; several studies indicated that normalization could have a substantial effect on the extracted solution.	Strong	Filtering and normalization parameters should be fully reported and consistently applied. No single preprocessing configuration should be assumed to be optimal across tasks and datasets.
	[28,155,157].	Simulation and human experimental studies	Multidirectional reaching, point-to-point reaching, MVIC/MVIE trunk tasks.	Number of trials, task directions, data concatenation, and task definition.	VAF, R^2^, number of synergies.	Insufficient task sampling can alter the extracted synergy structure; concatenation produced more synergies than averaging in reaching, and different trunk tasks required different numbers of synergies.	Moderate	Experimental tasks should provide sufficient repetitions and movement/task variability. Trial-processing strategies such as concatenation should be explicitly reported because they can affect estimated model order.
	—	—	—	Minimum reporting standards and open data.	Transparency, reproducibility, cross-study comparability.	The current evidence base demonstrates substantial methodological heterogeneity, but the direct effect of reporting standards or open databases has not been experimentally quantified.	Indirect	Participant characteristics, tasks, electrode locations, acquisition systems, preprocessing, and analysis parameters should be reported in detail. Open raw/processed EMG datasets should be regarded as a desirable direction for reproducibility rather than an empirically established requirement.
Synergy extraction and model selection	[35,59,60,105,141].	Algorithm development studies	Simulated and experimental EMG datasets/dynamic motor tasks.	Factorization algorithm.	Synergy recovery, VAF, repeatability, intra-subject consistency.	Performance differed according to algorithm, signal structure, channel number, and correlation among components. No single method consistently showed superiority for every criterion and dataset.	Moderate	Factorization methods should be selected according to data characteristics and study objectives rather than treating one algorithm as universally optimal.
	[135,149].	Algorithm development studies	Experimental/simulated EMG and upper-limb data.	Initialization strategy.	Reconstruction accuracy, convergence, synergy similarity.	Sparse initialization improved reconstruction in one methodological comparison, whereas autoencoder-derived solutions were reported to be sensitive to initialization.	Moderate	Initialization procedures should be reported and repeated, or alternative initializations should be considered when solution stability may be affected.
	[145,146,147,149].	Algorithm development studies	Simulated data, isometric reaching, reaching, upper-limb tasks.	Advanced factorization frameworks.	Force reconstruction, R^2^, VAF, RMSE, synergy similarity, interpretability.	Advanced methods improved particular aspects such as force reconstruction, nonlinear representation, or task-space integration, but higher reconstruction accuracy did not necessarily correspond to greater physiological interpretability.	Limited–Moderate	Bayesian, tensor, nonlinear, and deep/latent-variable approaches should be viewed as promising alternatives rather than universally superior replacements for conventional linear methods. Their interpretability, robustness, and computational requirements require further evaluation.
	[110,150,155,157].	Algorithm development and human experimental studies	EMG datasets, reaching, trunk MVIC/MVIE.	Model-order determination: VAF/R^2^, ΔVAF, modified AIC, trial-processing effects.	Selected number of synergies, VAF/R^2^, residual structure.	Modified AIC outperformed conventional threshold rules in the evaluated datasets; model order also changed with task type and data-processing strategy, while high explained variance did not guarantee that residual information was negligible.	Moderate	Model order should preferably be evaluated using complementary criteria rather than a single fixed VAF/R^2^ threshold. Threshold values should be interpreted in a task- and dataset-specific context.
	[110].	Algorithm development study	Multiple datasets under different SNR and filtering conditions.	Information-theoretic model selection.	Modified AIC and selected model order.	Modified AIC showed robust model-order estimation across the tested conditions; however, the current evidence base for AIC/BIC implementation in temporally autocorrelated EMG remains limited.	Indirect	When AIC, BIC, or related criteria are used, the assumed statistical model, parameter-counting scheme, and definition of effective sample size should be explicitly stated.
Validation and application oriented approaches	[17,27,37,40,117].	Human and clinical experimental studies	Upper-limb reaching, locomotor tasks, hand grasping, walking.	Reliability, repeatability, electrode placement, session effects.	Synergy similarity, VAF, number of synergies, temporal patterns.	Repeatability was variable across studies; electrode replacement and intrinsic EMG variability influenced reproducibility, while some synergy structures remained stable across sessions or days.	Moderate	Reliability and repeatability should be assessed whenever synergy stability is an important outcome. Electrode placement and between-session acquisition consistency require particular attention.
	[16,22,116,148,157].	Animal and human experimental studies	Postural perturbation, 3D force matching, constrained forearm tasks, basic/complex motor tasks, trunk MVIC/MVIE.	Task and experimental constraints.	Synergy composition, number of synergies, VAF, network structure.	Synergies can remain relatively stable within comparable task conditions, but their number, composition, or network organization may change with task type, force/position constraints, or task complexity.	Strong	Synergies should be interpreted as task-dependent representations; findings obtained from one task should not automatically be generalized to substantially different motor behaviors.
	[145,147,150].	Algorithm development and human experimental studies	Isometric reaching and reaching.	External biomechanical validation.	Force reconstruction, task-space variables, residual structure.	Synergy-based models showed meaningful relationships with force or kinematic/task-space variables, but unexplained residuals could still contain performance-relevant information.	Moderate	Reconstruction accuracy should be complemented by kinetic, kinematic, musculoskeletal-model, or other physiological validation when possible. Such agreement supports functional plausibility but does not independently prove a neural origin.
	[24,30,40].	Clinical studies	Walking and gait analysis.	Population-specific applicability.	VAF, variability, repeatability, synergy structure.	Clinical datasets showed that preprocessing and repeatability characteristics can differ substantially across subjects and conditions; direct evidence for universal cross-population generalization remains limited.	Indirect	Validate methods in the target task and population. Do not directly transfer settings from healthy participants to clinical populations.

## Data Availability

No new data were created or analyzed in this study. Data sharing is not applicable to this article.

## Abbreviations

The following abbreviations are used in this manuscript:

**Acronym**	**Muscle**	**Acronym**	**Muscle**
AL	Adductor Longus	ANC	Anconeus
AE	Abdominal Ext	MT	Middle Trapezius
BIC Long	Biceps Brachii Long Head	BIC Short	Biceps Brachii Short Head
BRD	Brachioradialis	BRA	Brachialis
BB	Biceps Brachii	BF	Biceps Femoris
BFMA	Anterior Biceps Femoris	BFMP	Posterior Biceps Femoris
CORB	Coracobrachialis	BFMM	Medial Biceps Femoris
DELT2	Medial Deltoid	DELT1	Anterior Deltoid
ECU	Extensor Carpi Ulnaris	DELT3	Posterior Deltoid
ECRL	Extensor Carpi Radialis Longus	ECR	Extensor Carpi Radialis
ECRB	Extensor Carpi Radialis Brevis	FDS	Flexor Digitorum Superficialis
FCR	Flexor Carpi Radialis	FCU	Flexor Carpi Ulnaris
ED	Extensor Digitorum	FPL	Flexor Pollicis Longus
PL	Peroneus Longus	PB	Peroneus Brevis
PT	Pronator Teres	PLAN	Plantaris
PECM	Pectoralis Major	PEC	Pectoralis
PECM3	Pectoralis Major Inferior	PECM1	Pectoralis Major Clavicular
PQ	Pronator Quadratus	PECM2	Pectoralis Major Medial
SO	Soleus	RF	Rectus Femoris
Sar	Sartorius	RI	Rectus Internus
MF	Multifidus	SRTM	Medial Sartorius
SUP	Supinator	SM	Semimembranosus
SUBSC	Subscapularis	ST	Semitendinosus
TRAPS	Superior Trapezius	SUPSP	Supraspinatus
TRI	Triceps Brachii	TRAPM	Middle Trapezius
TRI Long	Triceps Long Head	TRI Lat	Triceps Lateral Head
TMIN	Teres Minor	TRI Med	Triceps Medial Head
TA	Tibialis Anterior	TMAJ	Teres Major
GM	Gluteus Medius	TFL	Tensor Fasciae Latae
GLUA	Anterior Gluteus Medius	GLUP	Posterior Gluteus Medius
LAT	Latissimus Dorsi	LH	Lateral Hamstring
GL	Lateral Gastrocnemius	INFRA	Infraspinatus
LAT1	Latissimus Dorsi Superior	IO	Internal Oblique
IP	Iliopsoas	LAT2	Latissimus Dorsi Medial
MH	Medial Hamstring	LAT3	Latissimus Dorsi Inferior
VL	Vastus Lateralis	MG	Medial Gastrocnemius
VE	Vastus Externus	VM	Vastus Medialis
VI	Vastus Internus	SAN	Serratus Anterior
RHO	Rhomboid Major	ERS	Erector Spinae
IL3	Iliocostalis Lumborum at 3 cm	EO	External Oblique
GLU	Gluteus Maximus	IL6	Iliocostalis Lumborum at 6 cm
GAS	Gastrocnemius	RA	Rectus Abdominis
HAM	Hamstring	SCM	Sternocleidomastoid
VAS	Vasti	AM	Adductor Magnus
UT	Upper Trapezius	ESC	Erector Spinae (cervical region)
ESL	Erector Spinae (lumbar region)	EST	Erector Spinae (thoracic region)
ESL-Dom	Erector Spinae Longissimus dominant	ESL-Non	Erector Spinae Longissimus Non-dominant
GL	Gastrocnemius Lateralis	FD	Flexor Digitorum
LD1	Latissimus Dorsi thoracic	LD2	Latissimus Dorsi lumbar
EDL	Extensor Digitorum Longus	SEMA	Anterior Semimembranosus
ILPS	Iliopsoas	SEMP	Posterior Semimembranosus
GRAA	Anterior Gracilis	FDL	Flexor Digitorum Longus
GRAP	Posterior Gracilis	SRTA	Anterior Sartorius
RA	Rectus Abdominis	EDC	Extensor Digitorum Communis
EPL	Extensor Pollicis Longus	APL	Abductor Pollicis Longus
EPB	Extensor Pollicis Brevis	SPL	Splenius Capitis
ES	Erector Spinæ	LD3	Latissimus Dorsi iliac
MF-Dom	Multifidus, Dominant Side	MF-Non	Multifidus, Non-Dominant Side
LD-R	Right Longissimus Dorsi	LD-L	Left Longissimus Dorsi
